# Correlation between tooth decay and insulin resistance in normal weight males prompts a role for myo-inositol as a regenerative factor in dentistry and oral surgery: a feasibility study

**DOI:** 10.3389/fbioe.2024.1374135

**Published:** 2024-07-31

**Authors:** Fulvio Barbaro, Giusy Di Conza, Francesca Pia Quartulli, Enrico Quarantini, Marco Quarantini, Nicoletta Zini, Celine Fabbri, Salvatore Mosca, Silvio Caravelli, Massimiliano Mosca, Paolo Vescovi, Simone Sprio, Anna Tampieri, Roberto Toni

**Affiliations:** ^1^ Department of Medicine and Surgery - DIMEC, Laboratory of Regenerative Morphology and Bioartificial Structures (Re.Mo.Bio.S.), Museum and Historical Library of Biomedicine - BIOMED, University of Parma, Parma, Italy; ^2^ Odontostomatology Unit, and R&D Center for Artificial Intelligence in Biomedicine and Odontostomatology (A.I.B.O), Galliera Medical Center, San Venanzio di Galliera, Italy; ^3^ CNR Institute of Molecular Genetics “Luigi Luca Cavalli-Sforza”, Unit of Bologna, Bologna, Italy; ^4^ Course on Odontostomatology, University Vita-Salute San Raffaele, Milan, Italy; ^5^ Course on Disorders of the Locomotor System, Fellow Program in Orthopaedics and Traumatology, University Vita-Salute San Raffaele, Milan, Italy; ^6^ O.U. Orthopedics Bentivoglio, IRCCS Istituto Ortopedico Rizzoli, Bologna, Italy; ^7^ Department of Medicine and Surgery - DIMEC, Odontostomatology Section, University of Parma, Parma, Italy; ^8^ CNR - ISSMC, Faenza, Italy; ^9^ Academy of Sciences of the Institute of Bologna, Section IV - Medical Sciences, Bologna, Italy; ^10^ Endocrinology, Diabetes, and Nutrition Disorders Outpatient Clinic - OSTEONET (Osteoporosis, Nutrition, Endocrinology, and Innovative Therapies) and R&D Center A.I.B.O, Centro Medico Galliera, San Venanzio di Galliera, Italy; ^11^ Division of Endocrinology, Diabetes, and Metabolism, Department of Medicine, Tufts Medical Center - Tufts University School of Medicine, Boston, MA, United States

**Keywords:** tooth decay, caries, body composition, energy balance, insulin resistance, myo-inositol, endothelial cell, mesenchymal stromal cell

## Abstract

**Background:**

In an era of precision and stratified medicine, homogeneity in population-based cohorts, stringent causative entry, and pattern analysis of datasets are key elements to investigate medical treatments. Adhering to these principles, we collected *in vivo* and *in vitro* data pointing to an insulin-sensitizing/insulin-mimetic effect of myo-inositol (MYO) relevant to cell regeneration in dentistry and oral surgery. Confirmation of this possibility was obtained by *in silico* analysis of the relation between *in vivo* and *in vitro* results (the so-called bed-to-benchside reverse translational approach).

**Results:**

Fourteen subjects over the 266 screened were young adult, normal weight, euglycemic, sedentary males having normal appetite, free diet, with a regular three-times-a-day eating schedule, standard dental hygiene, and negligible malocclusion/enamel defects. Occlusal caries were detected by fluorescence videoscanning, whereas body composition and energy balance were estimated with plicometry, predictive equations, and handgrip. Statistically significant correlations (Pearson r coefficient) were found between the number of occlusal caries and anthropometric indexes predicting insulin resistance (IR) in relation to the abdominal/visceral fat mass, fat-free mass, muscular strength, and energy expenditure adjusted to the fat and muscle stores. This indicated a role for IR in affecting dentin reparative processes. Consistently, *in vitro* administration of MYO to HUVEC and Swiss NIH3T3 cells in concentrations corresponding to those administered *in vivo* to reduce IR resulted in statistically significant cell replication (ANOVA/Turkey tests), suggesting that MYO has the potential to counteract inhibitory effects of IR on dental vascular and stromal cells turnover. Finally, in *in silico* experiments, quantitative evaluation (WOE and information value) of a bioinformatic Clinical Outcome Pathway confirmed that *in vitro* trophic effects of MYO could be transferred *in vivo* with high predictability, providing robust credence of its efficacy for oral health.

**Conclusion:**

Our reverse bed-to-benchside data indicate that MYO might antagonize the detrimental effects of IR on tooth decay. This provides feasibility for clinical studies on MYO as a regenerative factor in dentistry and oral surgery, including dysmetabolic/aging conditions, bone reconstruction in oral destructive/necrotic disorders, dental implants, and for empowering the efficacy of a number of tissue engineering methodologies in dentistry and oral surgery.

## 1 Introduction

Ideal treatments in regenerative dentistry, oral surgery, and dental/oral tissue engineering are variably based on hard tissues regulatory factors, enhanced vascularization, biomaterial scaffolds, and cells of the dental–alveolar–maxillomandibular complex (Yao et al., 2020; Cao et al., 2021; Vaquette et al., 2022; Wang et al., 2022), such as pulpar, apical, periodontal, and alveolar stem cell (Sloan and Smith, 2007; Huang et al., 2010; Demarco et al., 2011; Moreno Sancho et al., 2019; Mozaffari et al., 2019; Ahmed et al., 2020; Fu et al., 2022; Guy, 2022; Jafer et al., 2022; Li et al., 2023). Interestingly, pulpar stem cells and odontoblasts may be mobilized by factors activating the phosphatidylinositol 3 kinase (PI3K)/Akt/mTOR and Wnt/beta catenin signaling pathways, leading to the formation of reactionary/reparative dentin (Li et al., 2017; Neves et al., 2017; Neves and Sharpe, 2018). As these transduction cascades have the potential to cross-talk to induce a mitogenic response of neuroectoderm-derived cells (Precilla et al., 2022), it would seem ideal to investigate molecules favoring this cross-talk in ectomesenchymal cells of the dental pulp and/or apical papilla (Demarco et al., 2011; Mozaffari et al., 2019). Stem cells from the periodontal ligament and mesenchymal stromal cells (MSCs) of the alveolar and masticatory bones might equally respond to this strategy, with regeneration of the alveolar and surrounding bone tissue, including enhanced cell homing (Kim et al., 2010; Ramazzotti et al., 2019; Zhang et al., 2020; Fu et al., 2022).

One of these growth molecules might be the nutritional supplement myo-inositol (MYO). After uptake through a sodium-dependent active transport at the plasma membrane (Cotlier, 1970; Spector and Lorenzo, 1975; Greene and Lattimer, 1982; Gillon et al., 1983; Segal et al., 1984; Biden and Wollheim, 1986; Yorek et al., 1986; Khatami and Rockey, 1988; Nakanishi et al., 1989; Yorek and Dunlap, 1989; Haneda et al., 1990; Okuda et al., 1991), MYO accumulates intracellularly 5–500 times more than in the plasma and extracellular fluids (Dawson and Freinkel, 1961; Lewin et al., 1976). In the cell, MYO may interact with cytidine diphosphate diacylglycerol (CDP-DAG) in a way that through a CDP-DAG inositol phosphatidyl transferase, phosphatidylinositol (PtdIns) is formed. Then, PtdIns is enzymatically converted into phosphatidylinositol-(4)-monophosphate (PtdIns4P) and phosphatidylinositol-(4,5)-bisphosphate (PtdIns4,5P2). PtdIns4,5P2, located in the inner portion of the plasma membrane, is the primary substrate responding to receptor tyrosine kinases (RTKs) (Michell et al., 1981; Berridge et al., 1983). In turn, RTKs may recruit PI3K to convert PtdIns4,5P2 into phosphatidylinositol-3,4,5-triphosphate (PIP3), triggering the Akt/mTOR signaling pathway (Simioni et al., 2018; Tuncel and Kalkan, 2018), which is interfaceable with the Wnt/beta catenin cascade (Precilla et al., 2022). MYO may also activate the inositol phosphate multikinase–energy sensor AMPK duo (Cabrera-Cruz et al., 2020) that, in turn, may impinge onto both the Akt/mTOR and Wnt/beta catenin transduction chains (Chen H et al., 2014), which is a finding that is consistent with the role of AMPK in inducing differentiation of dental pulp MSCs (Pantovic et al., 2012). Finally, MYO may activate the PI3K/Akt signaling switch independently on PIP3 through the inositol phosphoglycans-PP2Calpha signaling (Bevilaqua and Bizzarri, 2018; Lopez-Gambero et al., 2020). Thus, MYO can fulfill the requirement for a mitogenic activator of different types of dental–alveolar and surrounding bone cells.

Intriguingly, the PI3K/Akt/mTOR signaling pathway is a key switch for insulin, and it is inactivated in insulin resistance (IR) (Li et al., 2022; Ramasubbu and Devi Rajeswari, 2023). As MYO may act as an insulin sensitizer/insulin mimetic by re-activation of the PI3K/Akt/mTOR transduction cascade (Bevilacqua and Bizzarri, 2018; Cabrera-Cruz et al., 2020), we reasoned that *in vivo* clinical conditions of IR might benefit from MYO to restore the PI3K/Akt/mTOR growth-promoting actions on dental and alveolar bone cells. In particular, vascular endothelial cells and fibroblasts/MSCs could be the primary targets. Indeed, they represent the largest pulpar cell populations (Gaje and Ceausu, 2020; Alvarez-Vasquez and Castaneda-Alvarado, 2022; Ren et al., 2022); both have very similar molecular signatures in pulp and periodontium, including alveolar bone (Pagella et al., 2021), and they respond to insulin and insulin-growth factors (IGFs) through the same receptor (Wang et al., 2012; Lauritano et al., 2015; Escudero et al., 2017; Oyanagi et al., 2019; Bashir, 2021; He et al., 2022; Huseynova et al., 2022), which also occurs in the course of dental caries (Alkharobi et al., 2018). Finally, activation of the PI3K/Akt signaling chains leads to their mobilization, renewal, and differentiation, and it is accompanied by the formation of mineralized dentin (Jiang et al., 2003; Tanaka et al., 2018; Budi et al., 2019; Ramazzotti et al., 2019; Zhang et al., 2020). Consistently, conditions of IR and hyperglycemia such as diabetes type 2 (DM2) and unhealthy obesity are associated with the interference of the PI3K/Akt/mTOR pathway (Mikami et al., 2022; Saghiri et al., 2022; Ramasubbu and Devi Rajeswari, 2023), leading to periodontal inflammation and cariogenic oral microbiota (Loyola-Rodriguez et al., 2011; Gurav, 2012; Prince et al., 2023). However, even a subtle IR without hyperglycemia may increase the risk of tooth decay (Demmer et al., 2017), likely by affecting the endothelium of the pulpar terminal circulation and fibroblasts of the dental pulp and alveolar bone, to give rise to a dental/periodontal microangiopathy with fibrosis (Bender and Bender, 2003; Puscasu et al., 2021).

Having this target in mind, we searched for dental caries in young euglycemic males without clinical, nutritional, and behavioral conditions predisposing to tooth decay but having a number of anthropometric indexes that predicted IR, as expected to occur in metabolically unhealthy normal weight (MUHNW) subjects (Cembrowska et al., 2016; Mathew et al., 2016; Stefan et al., 2017; Schulze, 2019; Stefan, 2020; Valderrabano et al., 2023). Confirmation of this relation suggested a key role of IR *per se* in their dental caries, and it prompted us to *in vitro* test MYO at *in vivo* concentrations on human umbilical vein endothelial cells (HUVECs) and mouse embryonic fibroblasts (Swiss NIH 3T3), which were chosen as recognized surrogates of vascular cells and fibroblasts/MSCs of the dental pulp and alveolar bone (Huang et al., 2006; Sun et al., 2011; Dissanayaka et al., 2012; Teng et al., 2012; Dissanayaka et al., 2015). Their *in vitro* growth response provided a proof-of-concept of the MYO potential to *in vivo* counteract detrimental effects of IR on the integrity of teeth and surrounding bone. Finally, based on an *in silico* simulation of published *in vitro* and *in vivo* trophic effects of MYO, including our own results, we yielded a Clinical Outcome Pathway (Korn et al., 2022) whose MYO regenerative actions resulted in high predictability based on the weight of evidence and information value (Spinu et al., 2020), making positive dental/alveolar outcomes by MYO a reasonable expectation. In summary, we aimed at providing evidence that MYO has the potential to be a regenerative factor in dentistry, oral surgery, and related tissue engineering. Consistently, we found a correlation between IR and the number of dental caries, a mitogenic effect of MYO on surrogates of dental cells such as endothelial cells and fibroblasts, and informatic evidence that these two results were causally linked through insulin signaling.

## 2 Materials and methods

### 2.1 *In vivo* study

#### 2.1.1 Human subjects and cross-sectional study

A pilot cross-sectional study was performed, based on 14 young male volunteers. The study sample was extracted from a population screening (266 Caucasian subjects) participating in the ORTODENT cross-sectional trial promoted by the Municipality of the town of Galliera, Italy (see at https://www.centromedicogalliera.com/progettoosteonet-comunedigalliera-universit%C3%A0diparma), aimed at evaluating the dental status in relation to the development of mouth tumors and oral bone mass density. The study was conducted at the Centro Medico Galliera (CMG) and developed under the tenure of the clinical research Collaboration Agreement 2020–2023 between CMG and the Department of Medicine and Surgery (DIMEC) of the University of Parma (UNIPR), Parma, Italy, entitled: *Outpatient Diagnostic-Therapeutic Quality and National and International Guidelines on Endocrine-Metabolic Disorders*.

All subjects were enrolled according to the EU Rules 2016/679, no single patient information was extracted in relation to a provision by the National (SSN)/Regional (SSR) HealthCare System of Italy, all patients received an informative report on the purposes of the clinical study, and all signed an informed consent for voluntary participation. No personal/sensitive data were included in the study, complete anonymity and confidentiality was ensured for each participant, and all the original documents were kept in an authorized repository at the CMG Health Center (see above) where the study took place, under the responsibility of the CMG legal representative who also supervised the ethical adequacy of the procedures adopted, the latter based on the NIH-USA Guiding Principles for Ethical Research (see at: https://www.nih.gov/health-information/nih-clinical-research-trials-you/guiding-principles-ethical-research). All data were collected in an outpatient setting, and a detailed history of each subject was collected. The inclusion criteria were male sex, aged 15–45 years, normal euglycemic laboratory profile, absence of clinical disturbances and/or chronic therapy, normal appetite, free diet, eating three times a day, low level of daily physical activity, and regular tooth brushing.

#### 2.1.2 Anthropometric evaluation

For anthropometric evaluation, measurements included body weight, waist circumference (WC—midpoint between the limit of the rib arch and the iliac crest), hip circumference (most protruding trochanteric point of both inferior limbs), body mass index (BMI = kg/m^2^), waist-to-hip ratio (WHR), waist-to-height ratio (WHtR), body roundness index or BRI (Thomas et al., 2013), a body shape index or ABSI (Krakauer and Krakauer, 2012), conicity index (CI) (Valdez, 1991), and fat mass (FM) percentage (%) or FM%. The latter was determined on the right side of the body, using a four skinfold plicometry (bicipital, tricipital, subscapular, suprailiac folds—three repetitions/each) and a Harpenden plicometer C-136. Then, age- and sex-related Durnin and Womersley formulas for body density (Durnin and Womersley, 1974) and, finally, Siri formula for % body fat = [(495/body density) - 450] were used for the conversion of body density to FM%. Finally, FM% was converted to a continuous variable by arcsine transformation.

Fat Free Mass (FFM) was calculated by the conversion of FM% to kg, through proportion with body weight, and obtained in kg by subtraction. Then, an FFM index (FFMI) was achieved by dividing FFM by squared height (kg/m^2^). Skeletal muscle strength was measured using a Jamar hydraulic hand dynamometer handled with the dominant hand. Each participant was in a seated position with the arm flexed at 90°, and they were asked to squeeze the dynamometer three times, each time at the maximum strength for 3 s, allowing to rest for 15 s between each measurement. The resulting handgrip value was taken as the average of these three repetitions. Then, handgrip was corrected by body weight to yield normalized grip strength (NGS). In addition, using the male-related Harris–Benedict formula, we calculated the resting energy expenditure (REE) as follows: REE = 66.5 + [(13.75 × weight in kg) + (5.003 × height in cm) - (6.75 × age)]. Total energy expenditure (TEE) was estimated as REE × 1.45 physical activity level (PAL), corresponding to a sedentary lifestyle (EFSA NDA Panel, 2013). REE and TEE were also adjusted to visceral fat indexes (BMI, WC, and BRI) and skeletal muscle mass and activity (FFMI and handgrip strength).

Finally, in analogy to the recently proposed composite metabolic syndrome score (Garcia-Hermoso et al., 2020), a composite anthropometry-dependent insulin resistance (IR) score (ADIRs) was calculated as the sum of the anthropometric indexes of each individual adjusted to the standard deviation (SD) of the sample, as follows: ADIRs = BMI/SD + WC/SD + WHR/SD + WHtR/SD + FM% (after arcsine transformation)/SD + BRI/SD + ABSI/SD + CI/SD + FFMI/SD + NGS/SD.

#### 2.1.3 Dental evaluation

For dental evaluation, the presence, distribution, and extension of dental plaque was studied by visual inspection and, when necessary, enforced by the plaque disclosing solution MIRA-2-TON and categorized using the plaque index by Silness and Loe (Silness and Loe, 1964). Occlusal caries were assessed by computer-assisted fluorescence videoscanning (VistaCam, Durr Dental) and counted referring to the WHO/FDI full-mouth method targeting teeth (n) in the right (1n–2n) and left (3n–4n) hemiportions of the jaw and maxilla, respectively. For each patient, malocclusion was analyzed according to the Angle classification (classes I to III), thresholds for overbite (>4 mm) and overjet (>2 mm) were selected as an average of the current published values (Kinaan, 1986), enamel disturbances of interest included opacities and hypoplasia/altered enamel color (Seow, 2014), fillings and/or missing teeth were recorded separately (if present), and a mean number of occlusal caries/subject was calculated.

### 2.2 *In vitro* study

#### 2.2.1 Cell lines, culture media, and myo-inositol (MYO) preparation

Human umbilical vein endothelial cells (HUVECs, donated by Dr. Silvia Panseri) and murine embryonic fibroblasts (Swiss NIH 3T3, donated by Prof. Lucio Cocco) were used. HUVECs were grown in vascular cell basal medium (VCBM) completed with the Endothelial Cell Growth Kit-VEGF containing the following: 5 ng/mL rhVEGF, 5 ng/mL rhEGF, 5 ng/mL rhFGF basic, 15 ng/mL rhIGF-1, 50 μg/mL ascorbic acid, 10 mM L-glutamine, 0.75 Units/mL heparin sulfate, 1 μg/mL hydrocortisone hemisuccinate, and 2% fetal bovine serum (FBS). The culture medium was complemented with 1% penicillin/streptomycin (P/S) and 20 mL of 50 mg/mL gentamicin.

Swiss NIH3T3 fibroblasts were cultured in DMEM-LG with the addition of either 10% or 5% FBS, depending on the experimental steps (see below 2.2.2), 1% P/S, 1% essential amino acids, 1% glutamine, and 20 mL of 50 mg/mL gentamicin.

A 15 mg/mL stock solution of MYO (Sigma Aldrich I7508; molecular mass 180.16 g/mol; solubility 50 ng/mL) was prepared in sterile water. Then, the solution was filtered with a sterile syringe equipped with a 0.22-microns filter under a biological laminar flow hood and used for dose–response curves with a 1:2 dilution procedure. Morphology and distribution of HUVECs and Swiss NIH3T3 fibroblasts during culture and in relation to the experimental challenge (see 2.2.2) were assessed using a Leica inverted Led DMi1 light microscope (LM) equipped with a proprietary digital camera.

#### 2.2.2 Dose–response curves of HUVECs and Swiss NIH3T3 fibroblasts to MYO, and light microscopic evaluation

HUVECs at passage 3 were seeded at a density of 5,000 cells/cm^2^ in a T75 flask and let to rest for 24 h; then, the medium was changed every 48 h up to culture subconfluence (80% occupancy of the plate surface). In the second step, cells were trypsinized with trypsin-EDTA 1% in PBS, centrifuged at 150 *g* x 5 min, resuspended in complete VCBM (see 2.2.1), reseeded at 5,000 cells/cm^2^ in a T75 flask, and let to grow up to passage 5. In the third step, these cells were seeded in a 96-multi-well plate at a density of 2,500 cells/cm^2^ using 200 µL of complete VCBM (see 2.2.1.), and serial concentrations of MYO (40–640 µM) were added to it and changed every 48 h. Controls received only the vehicle. Cells were stopped at 2, 4, 6, and 8 days of culture, the medium was removed, culture was washed with sterile DPBS, and the plates were either analyzed for cell morphology (see below) or used for DNA assay (see 2.2.3). Four replicates for each treatment and controls were made.

Swiss NIH3T3 fibroblasts were grown at subconfluence in a standard T75 flask using DMEM-HG with the addition of 10% FBS and 1% P/S. In the second step, cells were detached by trypsinization (see HUVEC protocol above), centrifuged at 130 *g* x 10 min, resuspended in completed DMEM-LG—5% FBS, and seeded at a density of 20,000 cells/cm^2^ in a 96-multi-well plate (200 mL of medium/well) with serial concentrations of MYO (40 microM–640 microM) added to the medium and changed every 48 h. Controls received only the vehicle. Using this strategy, the number of cells in each well at the end of the different incubation periods always remained below 250,000 cells, which was the threshold for DNA detection by CyQUANT (see 2.2.3.). Cells were stopped at 2, 4, and 6 days of culture, the medium was removed, culture was washed with DPBS to remove traces of phenol red present in the medium, and the plates were either analyzed for cell morphology or used for DNA assay (see 2.2.3). Four replicates for each treatment and controls were made.

Cell morphology was studied using a Leica inverted Led DM1 LM + digital camera, and cell monolayers were left unfixed, unstained, and adherent to the well. Optical enlargements ranged from x 5 to x 40.

#### 2.2.3 CyQUANT proliferation assay

To define the amount of DNA and, thus, the entity of cell replication in each sample, the CyQUANT^®^ Cell Proliferation Assay Kit (Invitrogen) was used. The CyQUANT dye solution was prepared by mixing the working buffer with the CyQUANT^®^ GR dye, both supplied by the kit. The concentrated cell-lysis buffer stock solution (component B) was diluted 20-fold in distilled water, and just prior to running the experiment, the CyQUANT^®^ GR stock solution (component A) was diluted into the 1X cell-lysis buffer. CyQUANT^®^ GR dye was used at a 2X final concentration for the experiment conducted on HUVECs to detect a number of cells between 50 and 100,000 cells, whereas a 5X final concentration was used for the experiment conducted on Swiss NIH3T3 fibroblasts to detect a number of cells between 50 and 250,000 cells. A reference standard curve for DNA was prepared in a 96-multi-well plate, as follows: 100 μg/mL bacteriophage lambda DNA (component C) was serially diluted (1:2) in CyQUANT^®^ GR/cell-lysis buffer to obtain DNA concentrations ranging from 1,000 to 62.5 ng/mL in a volume of 200 mL. For analysis, 5 mL of each digested sample (control and treated cells) were dissolved in 195 mL of CyQUANT dye solution, and three replicates were prepared for each sample. Three control replicates were made with the buffer. Analysis of the calibration curve and samples was performed using a Victor 3 V Multi-label Plate Reader (PerkinElmer) set to the fluorescein protocol (excitation 485 nm, emission 535 nm). Optical density values linearly increased with the DNA quantity, and the unknown DNA amount was inferred by interpolation with the reference curve. The quantity of DNA was expressed in ng/mL.

#### 2.2.4 Estimate of the size of the cellular sample, cell doubling, and doubling time

To obtain a reference standard curve to convert CyQUANT^®^ GR dye fluorescence values into approximate cell numbers, pellets with a fixed number of cells were prepared. HUVECs and Swiss NIH3T3 fibroblasts at passage 4 were grown to subconfluence in a T75 flask, trypsinized, centrifuged, and resuspended in the media, as described above. After cell count, 100,000 HUVECs and 400,000 Swiss NIH3T3 cells were transferred into a 1.5 mL Eppendorf vial and centrifuged (see 2.2.2) to obtain cell pellets. Pellets were washed with 1 mL of sterile DPBS and re-centrifuged, as described above. After removing the supernatant, dry pellets were resuspended by brief vortexing and, using CyQUANT^®^ GR dye/cell-lysis buffer, serial dilutions of each pellet were made in 200 mL to obtain a number of cells between 50,000–3,125 HUVECs and 200,000–12,500 Swiss NIH3T3. Optical density values linearly increased with the increase in the number of cells, and unknown cell numbers were inferred by interpolation with the reference curve. Controls consisted of only the buffer, and the curves were conducted in triplicate. Kinetic parameters of the experimental sample (controls and treated cultures) were obtained based on the following formulas:

$$\text{CD}=\ln\left(\text{Nf}/\text{ Ni}\right)\;/\;\ln2\text{ ; DT}=\text{CT }/\text{ CD},$$

where CD is the number of cell duplications, Nf and Ni are the final and initial numbers, respectively, of cells counted, DT is the doubling time, and CT is the cell culture time in days.

### 2.3 *In silico* study

#### 2.3.1 Bioinformatic construction of a quantitative clinical outcome pathway for MYO trophic effects

A quantitative clinical outcome pathway (COP) graph for MYO as a growth factor was constructed based on the toxicological concept of adverse outcome pathway or AOP (Spinu et al., 2020). Using the bioinformatic procedure available at the Wiki site https://aopwiki.org/, literature data from molecular to *in vivo* established growth-promoting effects of MYO were combined with our results, to obtain a robust theoretical frame for transferring *in vitro* to *in vivo* regenerative effects of MYO. Strength of prediction of the graph was provided by the weight of evidence (WOE) and information value (I.V.) of each node, expressing a rank of the categorical variable that links MYO signaling modality to its mitogenic activity.

In particular, WOE was computed as follows:

$$\text{ln}\left(\%\text{ of non}-\text{events }÷\;\%\text{ of events}\right),$$

where events and non-events corresponded to the number of positive and negative pieces of evidence for a specific categorical variable analyzed in the selected literature data (i.e., to the number of published papers proving or disproving/not including the MYO molecular/cellular/*in vivo* actions targeted by each node of the graph).

Consequently, I.V. was computed as follows:

$$\sum \left(\%\text{ of non}-\text{events}-\%\text{ of events}\right)*\text{WOE}.$$

The level of I.V. predictability was considered as weak (0.02–0.1), medium (>0.1–0.3), high (>0.3–0.5), and very high (>0.5). To compensate for a credibility bias possibly intrinsic to a very high I.V., an additional level of confidence was searched for the COP, based on the evidence for uninterrupted cause–effect relations from the molecular initiation event through the key events up to the clinical outcome, at progressively higher levels of biological organization and phylogenetic scale. This was achieved by searching through the dataset available at the Wiki site resource (see above) and databases of relevant literature accessible via Web of Science, PubMed, and Google Scholar up to November 2023.

### 2.4 Statistical analysis

In choosing a homogenous cohort (MUHNW patients) from a screened population, we used a stratification principle (males and specific age interval). Thus, only a small number of subjects were available for pattern recognition obtained by correlation analysis (Kumar and Chong, 2018). As a result, the sample size was equated to that of a prediction model with binary outcomes (yes/no). Therefore, we applied the rule of a minimum of 10 events/variable or 10EPV (Riley et al., 2020). With this sample size, a Pearson’s r effect size of 0.3 was predicted. Accordingly, correlation between the number of occlusal caries and parameters of body composition and energy expenditure (BMI, WC, WHR, WHtR, BRI, arcsin FM% or FM, ABSI, CI, REE/BMI, REE/WC, REE/BRI, TEE/BMI, TEE/WC, TEE/BRI, FMMI, handgrip strength, NGS, REE/FFMI, REE/handgrip strength, TEE/FFMI, and TEE/handgrip strength) as well as the composite IR score (ADIRs) were analyzed by linear regression and r correlation coefficient. In contrast, when comparing means of two groups, we assumed a normal distribution of data; thus, a small sample size of up to five items with independent measures was considered sufficient for a trustable effect size (de Winter 2013). Consistently, differences in a) the number of occlusal caries in relation to their anatomical position in the jaw and maxilla, b) CD, c) DT, and d) DNA content between the control and treated cell cultures were evaluated using a one-way analysis of variance (ANOVA) and *post hoc* Tukey’s HSD tests. All correlations and differences of means were considered statistically significant if *p* < 0.05.

## 3 Results

### 3.1 *In vivo* relation among tooth decay, body composition, and energy expenditure

The phenotypic and dental characteristics of our male sample are listed in Table 1. Note that truncal fat mass (FM%) and muscle strength adjusted to body weight or NGS had mean values compatible with a metabolically unhealthy normal weight (MUHNW) phenotype. In addition, a mean of 3.36 ± 4.45 caries/individual were recorded; in contrast, the distribution of caries in relation to the antimeric hemiportions of the jaw and maxilla was not statistically significant. Finally, the plaque index was irrelevant, and occlusal relations revealed absent/negligible malocclusion (21.4% of cases), overbite (7.1% of cases), overjet (21.4% of cases), and minor enamel dyschromia (42.9% of cases).

**TABLE 1:** Phenotypic characteristics of the 14 normal weight, young males studied.

Parameter	Mean ± SD
Age (years)	23.1 ± 9.5
Weight (kg)	70.3 ± 9.5
Height (cm)	174 ± 6.12
**Total body fat**	
BMI (kg/m^2^)	23.1 ± 2.6
FM%	25.4 ± 6.34
**Visceral fat**	
WC (cm)	81.6 ± 6.7
Hip circumference (cm)	95.1 ± 5.0
WHR	0.86 ± 0.04
WHtR	0.50 ± 0.04
BRI	2.74 ± 0.71
ABSI	0.0763 ± 0.0020
CI	1.18 ± 0.042
**FFM Muscle Mass**	
FFMI (Kg/m^2^)	17.19 ±2.49
Handgrip (kg)	39.52 ± 12.35
NGS (Kg/Kg weight)	0.56 ±0.13
**Energy expenditure**	
REE (Kcal/24 h)	1,747.77 ± 198.81
TEE (Kcal/24 h)	2,534.30 ± 198.81
**Insulin resistance**	
ADIRS	138.1 ± 5.92
**Occlusal caries**	
No caries/subject	3.36 ± 4.45
Right jaw (No.)	1 ± 1.30
Left jaw (No.)	0.86 ± 1.03
Right maxilla (No.)	0.93 ± 1.27
Left maxilla (No.)	0.79 ± 1.05^NS^

The large majority of anthropometric indexes were in the normal range; however, total fat mass (expressed as FM%) deduced by Durnin and Womersley trunk plicometry, and skeletal muscle strength adjusted to body weight or normalized grip strength (NGS) had mean values compatible with a metabolically unhealthy normal weight (MUHNW) phenotype. As expected in this phenotype by the presence of systemic insulin resistance (IR), a mean of more than three occlusal caries/subject were recorded; however, their distribution in the jaw and maxilla did not show any preferential anatomical location.

NS, not statistically significant vs all locations analyzed; No., number; ABSI, a body shape index; ADIRs, anthropometry-dependent insulin resistance score; BMI, body mass index; BRI, body roundness index; CI, conicity index; FM%, fat mass percentage; FFMI, fat-free mass index; REE, resting energy expenditure; TEE, total energy expenditure; SD, standard deviation; WC, waist circumference; WHR, waist-to-hip ratio; WHtR, waist-to-height ratio (data courtesy of Noemi Coppola, pre-doctoral fellow 2022–2023, Course of Human Nutrition Sciences, UNIPR, Parma, Italy).

**FIGURE 1:**
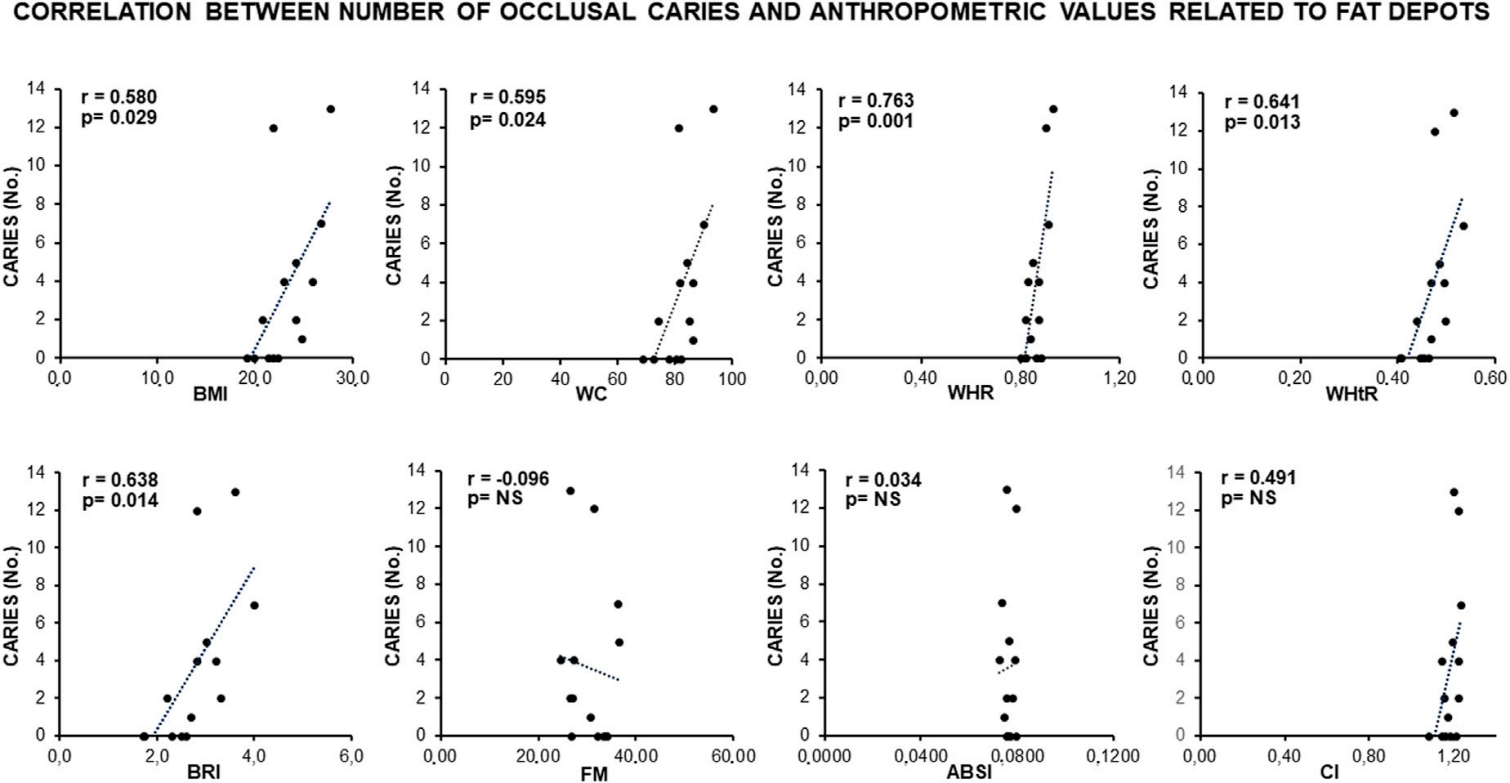
Correlation between the number of occlusal caries and anthropometric indexes of IR in the total sample of young males studied. Note a direct, statistically significant correlation involving >60% of indexes related to visceral fat (BMI, WC, WHR, WtHR, and BRI), suggesting that caries are related to a likely MUHNW phenotype. ABSI, a body shape index; BMI, body mass index; BRI, body roundness index; CI, conicity index; FM, fat mass percentage after arcsin transformation; WC, waist circumference; WHR, waist-to-hip ratio; WHtR, waist-to-height ratio; NS, not statistically significant (data courtesy of Noemi Coppola, pre-doctoral fellow 2022–2023, Course of Human Nutrition Sciences, UNIPR, Parma, Italy).

**FIGURE 2:**
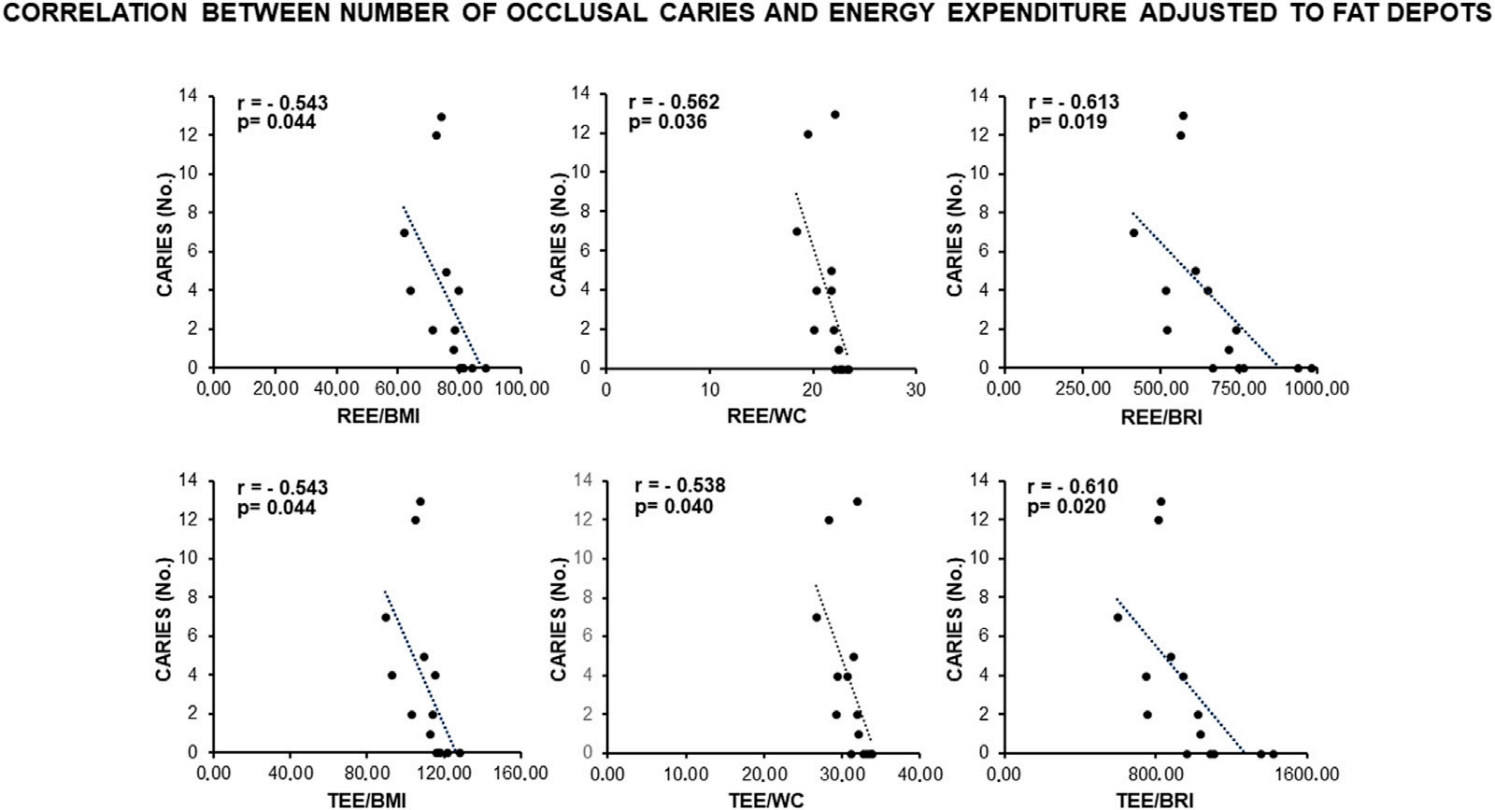
Correlation between the number of occlusal caries and energy expenditure adjusted to the anthropometric indexes of IR related to visceral fat in the total sample of young males studied. Note constancy of an inverse, statistically significant correlation supporting the association of caries with a likely MUHNW phenotype. BMI, body mass index; BRI, body roundness index; REE, resting energy expenditure; TEE, total energy expenditure; WC, waist circumference (data courtesy of Noemi Coppola, pre-doctoral fellow 2022–2023, Course of Human Nutrition Sciences, UNIPR, Parma, Italy).

Remarkably, a statistically significant association occurred between the number of occlusal caries and more than 60% of the anthropometric indexes related to visceral fat deposits (BMI, WC, WHR, WtHR, and BRI) (Figure 1). In addition, the number of occlusal caries displayed a statistically significant inverse correlation with the resting (REE) and total (TEE) energy expenditures adjusted to the indexes of visceral fat (BMI, WC, and BRI) (Figure 2). These data supported a MUHNW phenotype with IR, where a lower metabolic activity corresponded to a higher amount of visceral fat.

**FIGURE 3:**
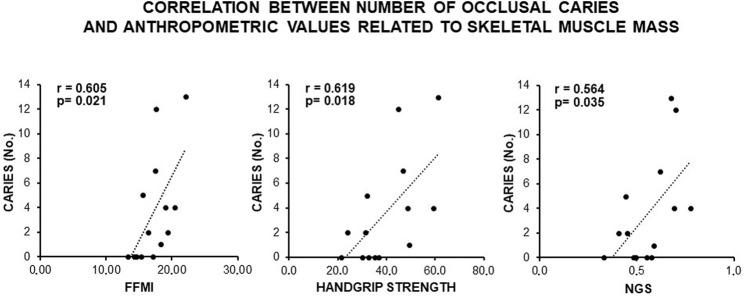
Correlation between the number of occlusal caries and anthropometric indexes of IR related to fat-free mass and skeletal muscle in the total sample of young males studied. Note a direct, statistically significant correlation suggesting contribution of the skeletal muscle mass to the likely MUHNW phenotype expected to lead to the recorded caries. FFMI, fat-free mass index; NGS, normalized grip strength (data courtesy of Noemi Coppola, pre-doctoral fellow 2022–2023, Course of Human Nutrition Sciences, UNIPR, Parma, Italy).

**FIGURE 4:**
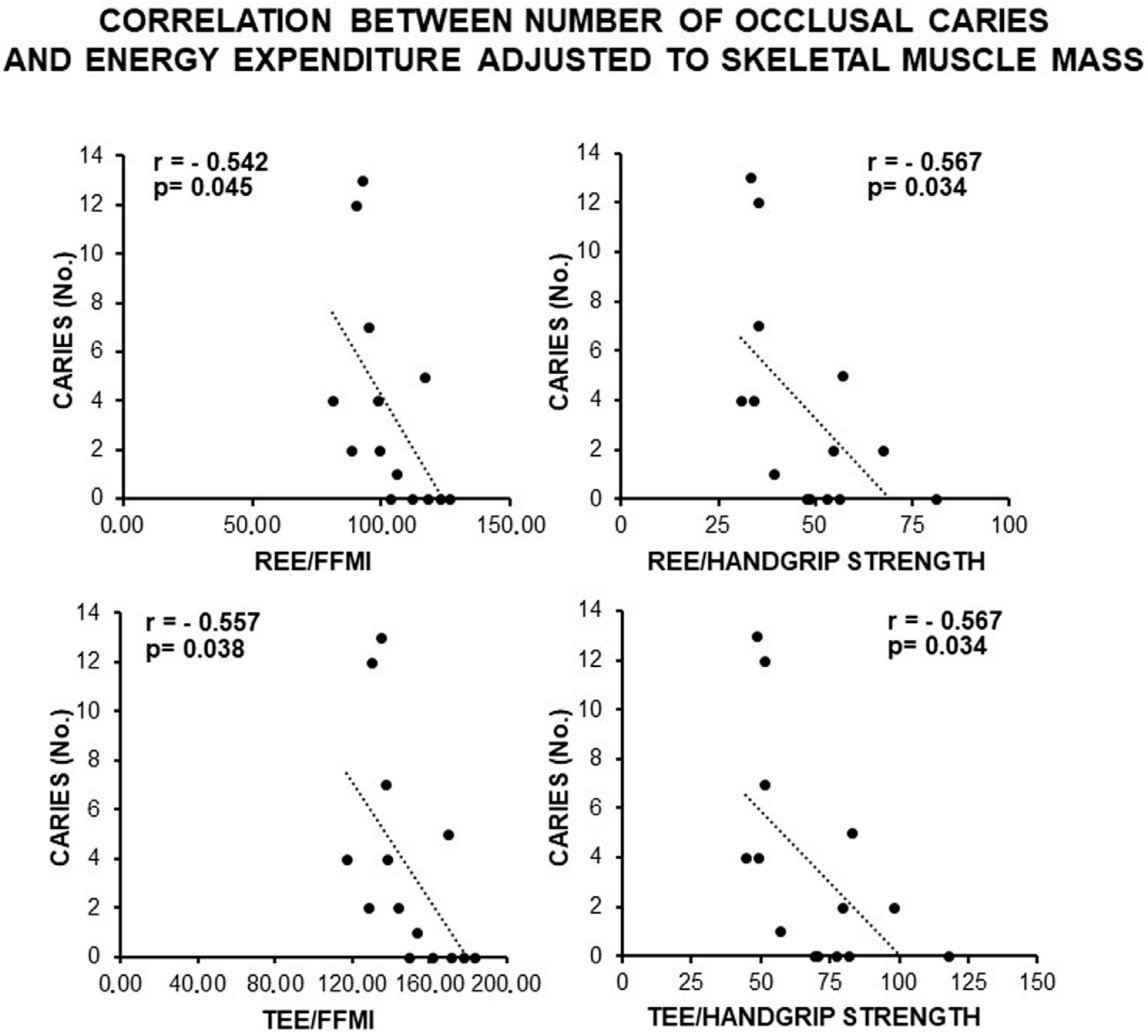
Correlation between the number of occlusal caries and energy expenditure adjusted to the anthropometric indexes of IR related to the skeletal muscle mass in the total sample of young males studied. Note constancy of an inverse, statistically significant correlation supporting the association of caries with a likely MUHNW phenotype. FFMI, fat-free mass index; REE, resting energy expenditure; TEE, total energy expenditure (data courtesy of Noemi Coppola, pre-doctoral fellow 2022–2023, Course of Human Nutrition Sciences, UNIPR, Parma, Italy).

Consistently, a direct and statistically significant correlation was observed between the number of occlusal caries and indexes of skeletal muscle mass (FFMI, handgrip, and NGS) (Figure 3), as well as an inverse statistically significant correlation was found when REE and TEE were adjusted to these indexes (Figure 4). This supported a MUHNW phenotype with IR, where a lower metabolic activity corresponded to a higher skeletal muscle mass.

**FIGURE 5:**
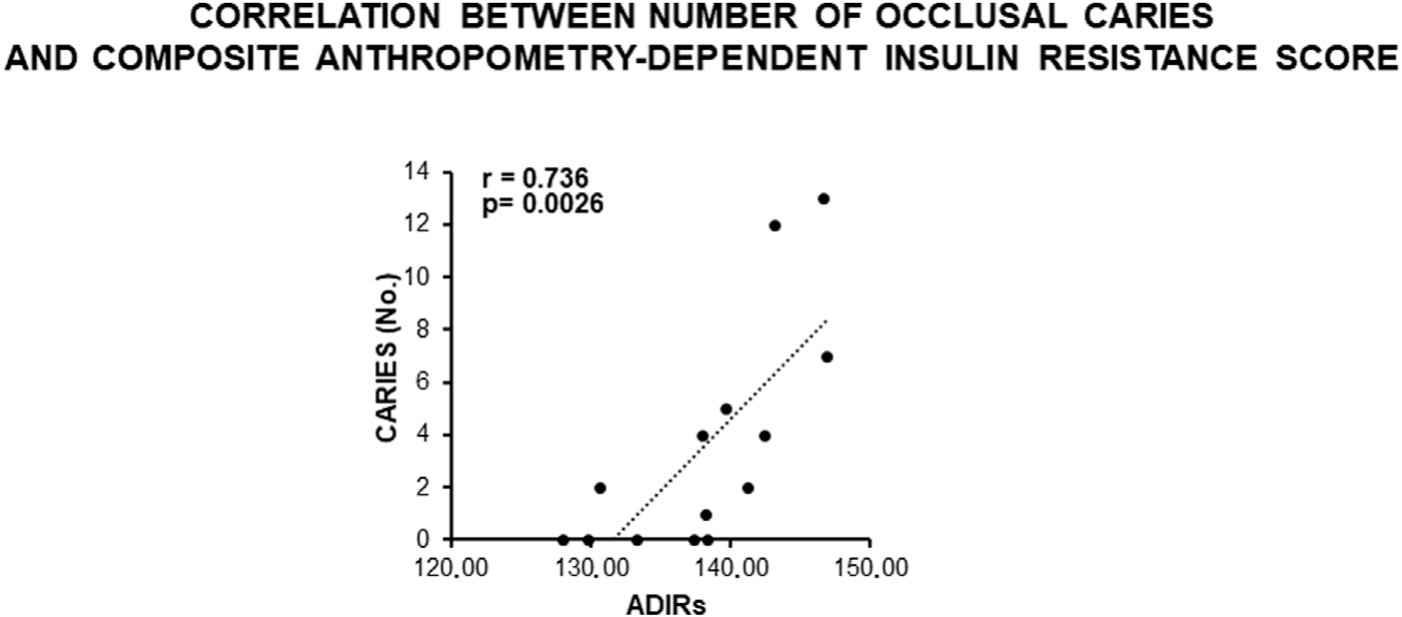
Correlation between the number of occlusal caries and composite anthropometry-dependent IR score in the total sample of young males studied. Note a direct, statistically significant correlation supporting a role of IR in the development of caries, as expected in MUHNW subjects. ADIRs, anthropometry-dependent IR score (data courtesy of Noemi Coppola, pre-doctoral fellow 2022–2023, Course of Human Nutrition Sciences, UNIPR, Parma, Italy).

Finally, a direct statistically significant correlation occurred between the number of occlusal caries and the composite score of IR, as deduced by the contribution of all anthropometric indexes related to fat and fat-free masses adjusted to the sample variance (Figure 5). This confirmed a MUHNW phenotype with IR as a likely causal factor for the cariogenic outcomes.

### 3.2 *In vitro* trophic effect of myo-inositol (MYO) on vascular and stromal cells

**FIGURE 6:**
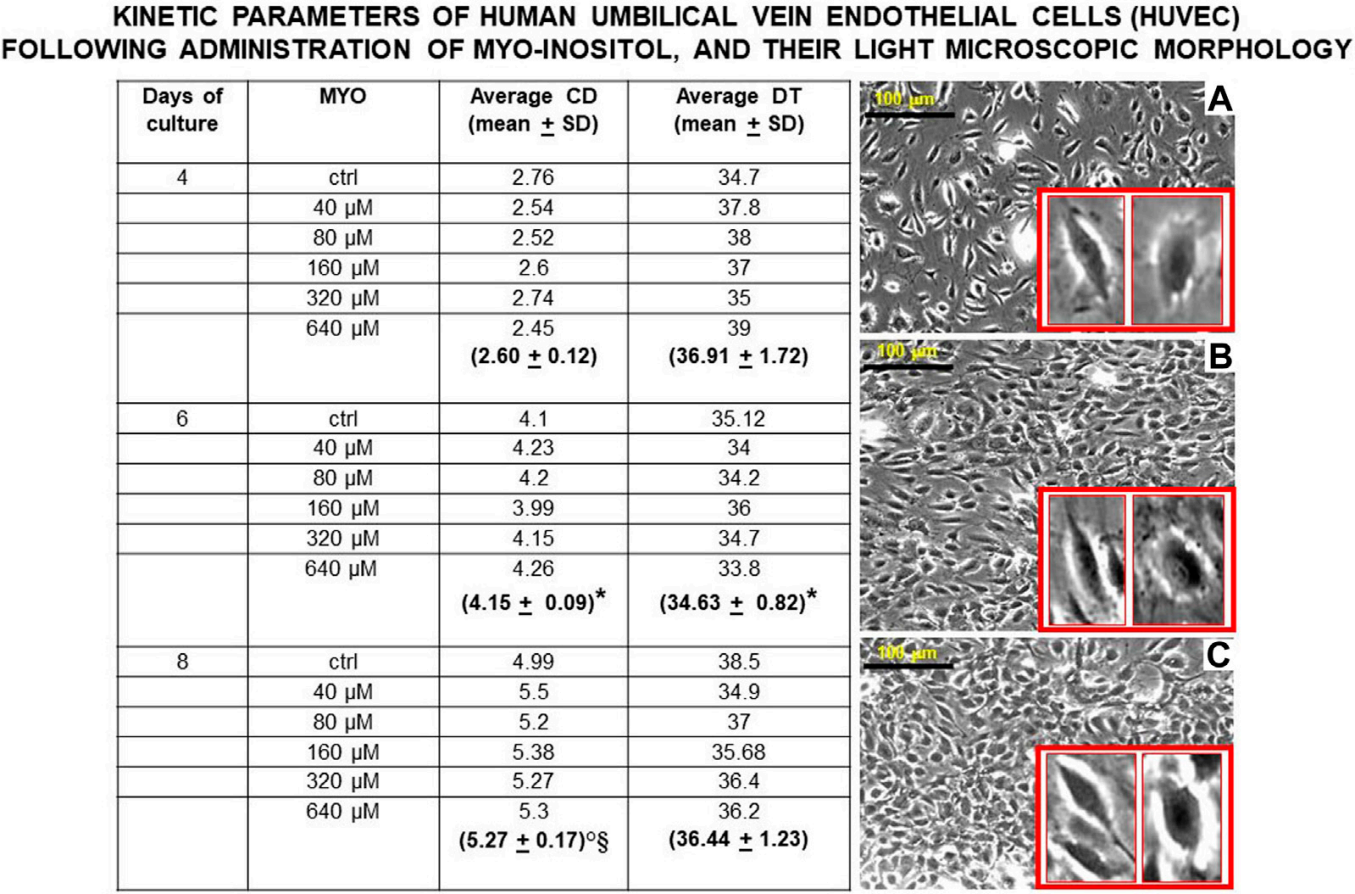
Values of kinetic parameters of human umbilical vein endothelial cells (HUVECs) in monolayer, surrogating endothelial cells of the dental pulp/oral bone. Following administration of different myo-inositol (MYO) concentrations, a statistically significant increase in cell doubling (CD) was observed at each time point (days 4, 6, and 8), suggesting a continuous recruitment of cells entering the cell cycle with all MYO amounts. Similarly, a statistically significant decrease in doubling time (DT) was detected during the first growth period (from days 4–6) with all MYO concentrations. In contrast, no statistically significant changes occurred to DT during the second growth period (from days 6–8), pointing to an initial spurt in cell growth velocity, which is expectedly stabilized by cell crowding and space-dependent constraints in the monolayer. Inverted light microscopy revealed that cells maintained constant spindle/polygonal morphology and integrity of the nuclear shape and structure for all 8 days of culture and with all MYO concentrations, suggesting the stability of the vascular endothelial phenotype. As a representative example of this morphological constancy independent on increased cell crowding in the cultures with MYO, we here show cell morphologies at day 8 **(A)** = control, **(B)** = 40 microM MYO, and **(C)** = 640 microM MYO (insets show enlargements of cell types present in each culture). Microscopy magnification ×5, marker = 100 microns. Each kinetic value and light microscopic image represents the average of four different experiments. Ctrl = control; * = *p* < 0.05 vs 4 days; ° = *p* < 0.05 vs 4 days; § = *p* < 0.05 vs 6 days (data courtesy of Sara Maioli, pre-doctoral fellow 2019–2020, Course of Medical, Veterinary, and Pharmaceutical Biotechnologies, UNIPR, Parma, Italy).

**FIGURE 7:**
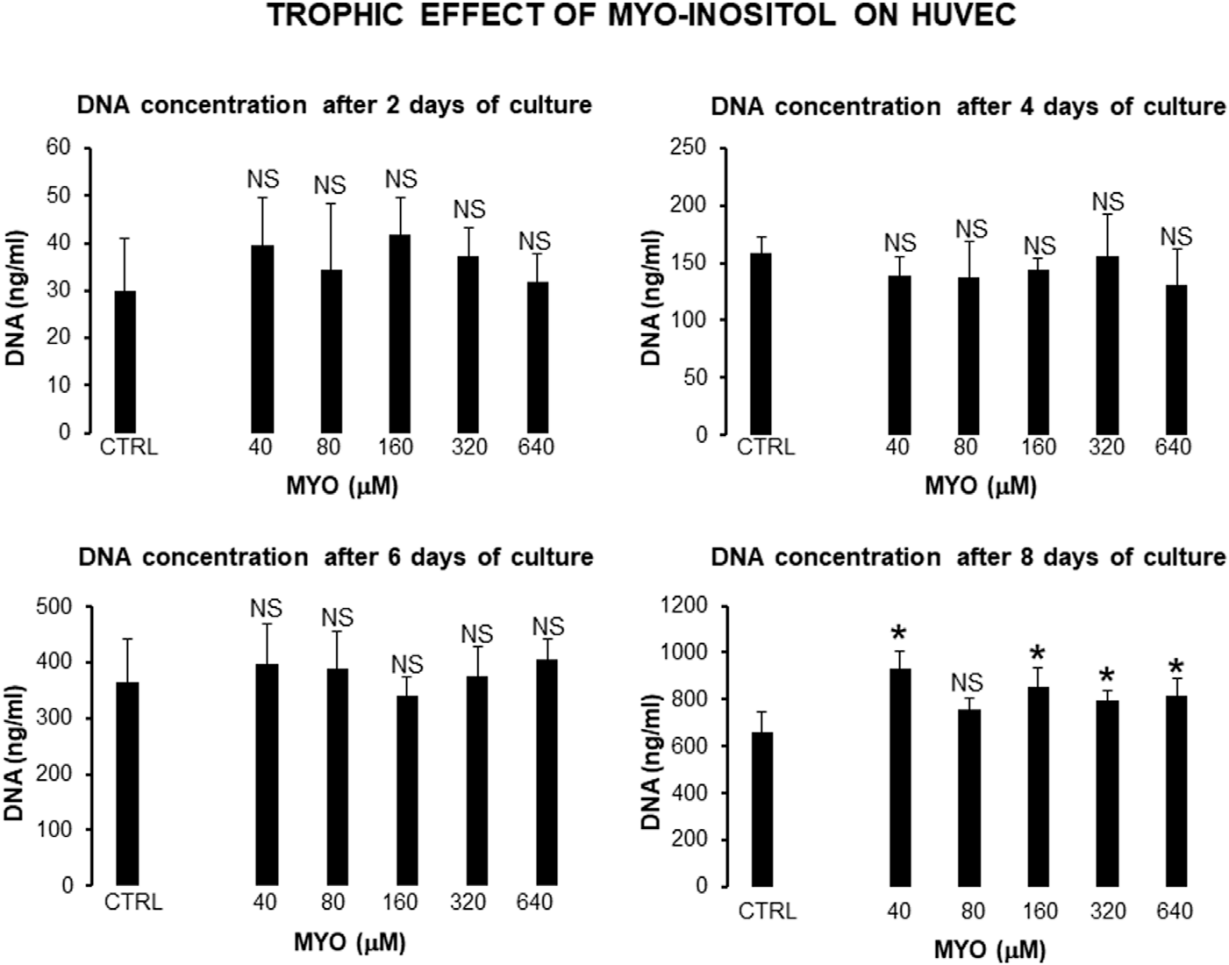
Effect of MYO administration to HUVECs in monolayer for 8 days. A statistically significant increase in DNA content of the culture occurred at day 8, with all but one (80 microM) MYO concentrations in respect to untreated controls. * = *p* < 0.05 treated vs untreated sample; NS = not statistically significant (data courtesy of Sara Maioli, pre-doctoral fellow 2019–2020, Course of Medical, Veterinary, and Pharmaceutical Biotechnologies, UNIPR, Parma, Italy).

Figure 6 shows the values of kinetic parameters of HUVECs in monolayer, following the administration of different MYO concentrations for 8 days. At each time point (days 4, 6, and 8) and with all MYO concentrations, we observed a statistically significant increase in cell doubling (CD). Similarly, we recorded a statistically significant decrease in doubling time (DT) during the first culture period (from days 4–6), which then rebalanced during the second culture period (from day 6–8). Cells maintained a stable endothelial morphology with all MYO concentrations for the entire period of culture. In particular, two main cell types were observed, including spindle/fibroblastoid and polygonal cells, with central nuclei without any visible nuclear fragmentation and/or atypia, and single/multiple nucleoli. Cytoplasm remained compact, without signs of shrinkage, apoptotic bodies, and/or plasma membrane blebs; however, plasmalemmal interdigitations were seen to connect adjacent cells. Consistent with kinetic data, Figure 7 shows that all but one (i.e., 80 microM) MYO concentrations significantly increased the DNA content of the culture at day 8 with respect to the untreated controls. Collectively, these results confirmed a direct regenerative effect of MYO on human vascular endothelial cells.

**FIGURE 8:**
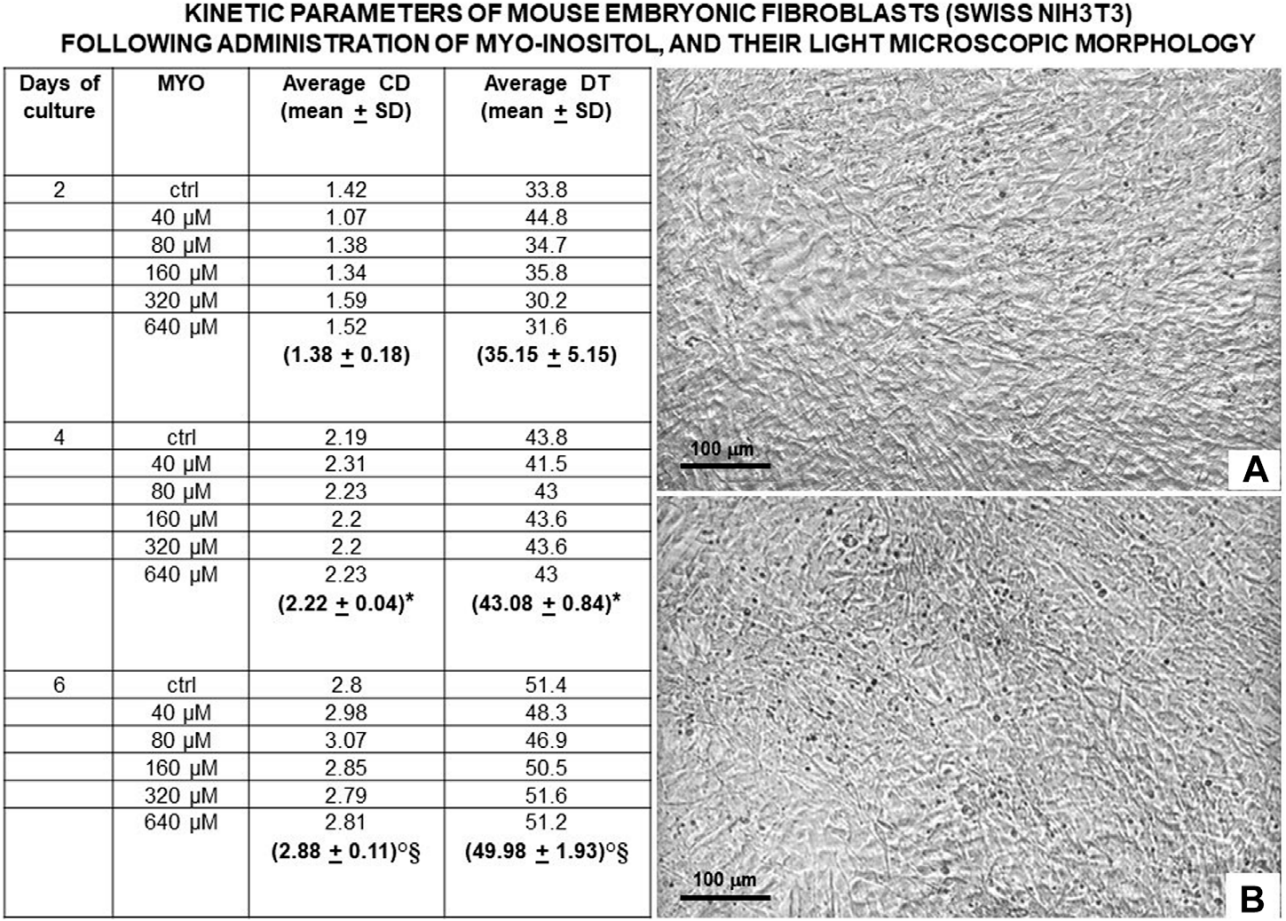
Values of kinetic parameters of mouse embryonic fibroblasts (Swiss NIH3T3) in monolayer, surrogating fibroblasts and mesenchymal stromal cells (MSCs) of the dental pulp/oral bone. Following administration of different MYO concentrations for 6 days, a statistically significant increase in CD was observed at each time point (days 2, 4, and 6) with all MYO concentrations, suggesting a continuous recruitment of cells entering the cell cycle. Similarly, a statistically significant increase in DT was detected during the entire period of culture (days 2, 4, and 6), pointing to a progressive deceleration in cell growth, an expected constraint from cell crowding in the monolayer. At day 6 of culture, inverted light microscopy revealed that control and MYO-stimulated embryonic fibroblasts gave rise to a very similar dense pavement of adherent elongated cells, suggesting the stability of their fibroblastic/MSCs-like phenotype for the entire period of culture; **(A)** = control and **(B)** = 80 microM MYO. Microscopy magnification ×5, marker = 100 microns. Each kinetic value and light microscopic image represents the average of four different experiments. Ctrl = control; * = *p* < 0.05 vs 4 days; ° = *p* < 0.05 vs 4 days; § = *p* < 0.05 vs 6 days (data courtesy of Sara Maioli, pre-doctoral fellow 2019–2020, Course of Medical, Veterinary, and Pharmaceutical Biotechnologies, UNIPR, Parma, Italy).

**FIGURE 9:**
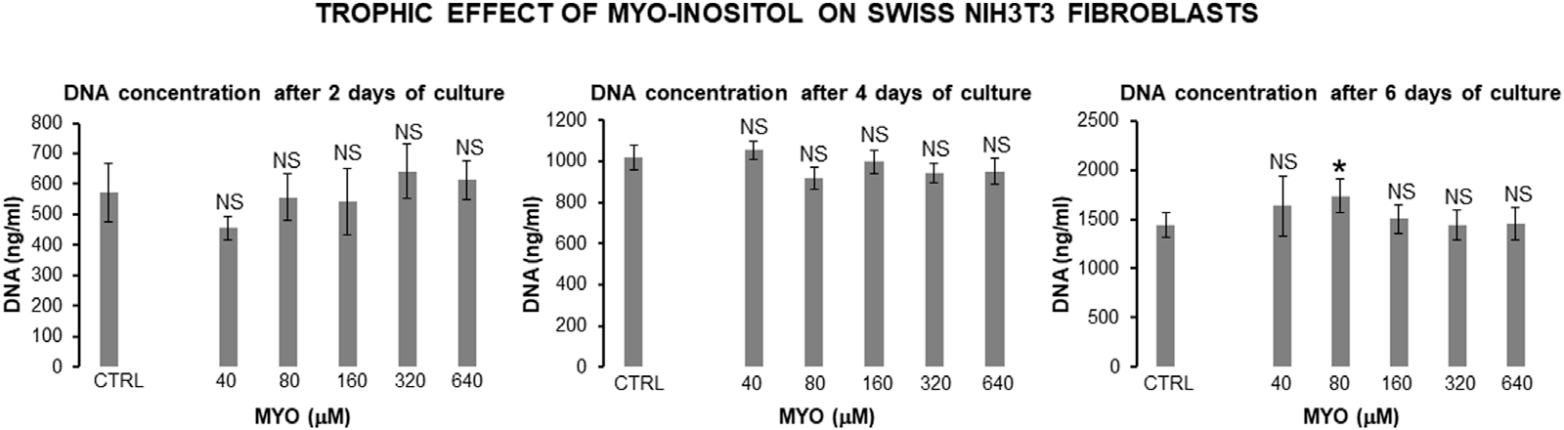
Effect of MYO administration to Swiss NIH3T3 cells in monolayer for 6 days. A statistically significant increase in DNA content of the culture occurred at day 6 with 80 microM MYO concentration in respect to untreated controls. * = *p* < 0.05 treated vs untreated sample; NS = not statistically significant (data courtesy of Sara Maioli, pre-doctoral fellow 2019–2020, Course of Medical, Veterinary, and Pharmaceutical Biotechnologies, UNIPR, Parma, Italy).

Figure 8 shows the values of kinetic parameters of the Swiss NIH3T3 cells in the monolayer, following the administration of different MYO concentrations for 6 days. At each time point (days 2, 4, and 6) and with all MYO concentrations, we observed a statistically significant increase in CD and DT. NIH3T3 cells maintained stable fibroblastic/MSC-like morphology with all MYO concentrations for the entire period of culture. However, only 80 microM MYO significantly increased the DNA content of the culture at day 6 with respect to the untreated controls (Figure 9), revealing a selective regenerative effect of MYO on mammalian fibroblastic/stromal cells.

### 3.3 *In silico* simulation of a linkage between *in vitro* and *in vivo* trophic effects of MYO

**FIGURE 10:**
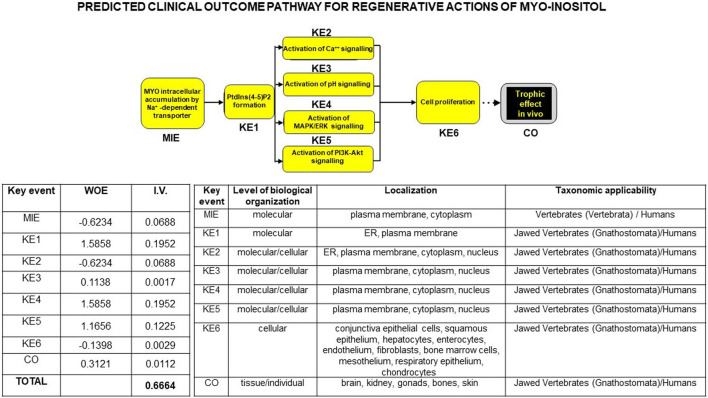
Flowchart of a bottom-up theoretical linkage or clinical outcome pathway (COP) for trophic/regenerative actions of MYO, bridging *in vitro* data to *in vivo* human evidence. Based on *ad hoc* selection of the international literature (75 publications), the knowledge chain was constructed using a procedure similar to that for adverse outcome pathways in toxicology (see at Wiki site https://aopwiki.org/). Strength of prediction in the ensuing bioinformatic linkage was provided by the weight of evidence (WOE) and information value (I.V.) expressing a rank of the categorical variable detailed inside each node (the descriptor of the MYO action at that level), and it is reported in the left table below the graph. Note that cumulated I.V. for MYO trophic actions was >0.5, indicating a very high predictive power of the cause–effect relations between the molecular initiation event (MIE) and subsequent measurable responses of MYO labeled as key events (KE) up to the final *in vivo* real-world evidence or clinical outcome (CO). Current experimental results contributed to the rank values of the categorical variables. A further threshold of credibility of the COP was provided by the spanning of cause–effect links through different levels of biological organization and taxonomic scale up to *Homo sapiens*, as summarized in the right table below the graph (data courtesy of Sara Maioli, pre-doctoral fellow 2019–2020, Course of Medical, Veterinary, and Pharmaceutical Biotechnologies, UNIPR, Parma, Italy).

Finally, to support a translational linkage between proven and published *in vitro* and *in vivo* trophic effects of MYO including our own data and substantiate the predictability of a regenerative action of MYO at the dental, alveolar, and oral bone levels, we bioinformatically developed a clinical outcome pathway for MYO trophic effects. As shown in Figure 10, we integrated current literature data on signaling/mitogenic/growth responses to MYO in different *in vitro* and *in vivo* models of cells/tissues/organs/animals/man with our current results. This led to the construction of a discrete (i.e., constrained by a specific direction of the information flow) bottom-up knowledge chain or graph comprising from the molecular to real-world levels. These different levels were linked by the cause–effect connections (or edges) of the selected parameters, with each parameter being the categorical variable targeting each node or descriptor of the MYO action at that level. Structurally, this graph was constructed based on a molecular initiating event through measurable responses of MYO at the cellular level or key events, up to the final evidence of growth of tissue/organs in animal models and humans or the clinical outcome.

Strength of prediction of the graph was provided by the weight of evidence and information value (I.V.) of each node, expressing a rank of each categorical variable linking MYO signaling modality to its mitogenic activity. Cumulated I.V. for all selected MYO actions yielded a very high predictive power (>0.5), which was unbiasedly corroborated by the notion that the cause–effect relations were constantly recordable throughout different levels of biological organization and phylogenetic scale, up to the human *in vivo* condition. Collectively, these results were strongly in favor of MYO as a regenerative factor, implying that it could be used to counteract the detrimental effect of IR on *in vivo* tooth decay.

## 4 Discussion

In this study, we applied the recently developed methodology of bed-to-benchside reverse translation analysis (Rudebeck et al., 2019) to investigate the role of the insulin sensitizer/insulin mimetic myo-inositol (MYO) as a new regenerative factor in dentistry and oral surgery. Based on the principles of *in vivo* stratified medicine and the analysis of homogenous patient clusters (Stengel et al., 2023), we found that *in vivo* insulin resistance (IR) correlated with the number of dental caries in metabolically unhealthy normal weight (MUHNW) subjects. In addition, *in vivo*-effective, insulin-sensitizing doses of MYO exerted *in vitro* a regenerative action on surrogates of dental/alveolar cells (endothelial cells and fibroblasts). Finally, these *in vivo* and *in vitro* results were causally linked through insulin signaling, whose MYO is a sensitizing/mimetic agent. Our data were corroborated by other experimental studies showing that MYO can *in vitro* enhance the replication of human vascular endothelial cells (Lorenzi and Toledo, 1986) and human skin fibroblasts (Sibbitt et al., 1989), whereas *in vivo*, it stimulates the growth of mouse mandibular chondrocytes (Yamaguchi et al., 2019), osteoblasts and osteoprogenitors (Dai et al., 2011), and bone calcium uptake (Angeloff et al., 1977). In the next sections, we will discuss in detail all these points.

### 4.1 *In vivo* clinical study

#### 4.1.1 Exclusion and inclusion criteria for the *in vivo* test of MYO as a new dental/alveolar regenerative factor

The sugar alcohol MYO has features compatible with a bioactive molecule in regenerative dentistry and oral surgery (Bermudez et al., 2021): it is easily deliverable both systemically and locally, is not toxic for long periods of administration, has a low cost, is compatible with the cell metabolism, and may act as a growth factor at key signaling steps to trigger replication of target cells, including the vascular endothelium (Lopez-Gambero et al., 2020). Clinically, it is used to reduce IR in disorders such as polycystic ovary syndrome (PCOS), diabetes mellitus type 2 (DM2), and gestational diabetes (for a review, see Unfer et al., 2012; Menichini and Facchinetti, 2017; Ozturan et al., 2019), which are all burdened by compromised dental/periodontal/alveolar integrity (Saghiri et al., 2021; Dou et al., 2023). In particular, DM2 patients develop periodontal inflammation and cariogenic oral microbiota (Garton and Ford, 2012), which is similar to that in patients with metabolically unhealthy obesity or MUHO (Suvan et al., 2011; Kim et al., 2020). This is a condition where the body mass index (BMI) is in the obesity range associated with hyperinsulinemia, hyperglycemia, dyslipidemia, hypertension, and cardiovascular manifestations, as opposed to the metabolically healthy obesity, where these complications are absent (Bluher, 2020). In all these disorders, IR and systemic inflammation are believed to be causative for tooth decay (Loyola-Rodriguez et al., 2011; Gurav, 2012; Prince et al., 2023). Surprisingly, no data are available on the role of MYO in counteracting development and/or favoring repair of dental caries and/or lesions of the periodontium and alveolar bone in either DM2 or MUHO. Thus, a clinical trial with MYO to counteract tooth decay would be advisable; however, it would imply specific requirements (Rajadhyaksha, 2010).

In particular, advanced glycated end products of DM2 interfere with the signaling systems activated by insulin and IGFs (Ramasubbu and Devi Rajeswari, 2023), leading to altered growth and turnover of dental/alveolar cells (Wang et al., 2012; Lauritano et al., 2015; Escudero et al., 2017; Oyanagi et al., 2019; Bashir, 2021; He et al., 2022; Huseynova et al., 2022), which also occurs in the course of dental caries (Alkharobi et al., 2018). Similarly, in PCOS and MUHO, unbalanced sex hormones and systemic inflammation can lead to a cariogenic microbiota (Menichini and Facchinetti, 2017; Saghiri et al., 2021). Thus, we focused on young euglycemic males of average healthy weight (BMI <25 kg/m^2^) without clinical, nutritional, and behavioral conditions, indicating a predisposition to dental caries, but harboring IR predicted on anthropometric bases, that is, in relation to the distribution of their fat and fat-free masses. These subjects have a metabolically unhealthy normal weight (MUHNW) phenotype, and they might represent elective responders to the dental effects of MYO.

#### 4.1.2 MUHNW males as a target group to investigate the potential of MYO in dental/alveolar health

MUHNW subjects are lean, but their fat distribution is primarily truncal, in particular, visceral/abdominal and ectopic. This may variably increase some anthropometric indexes such as waist circumference (WC), whereas their subcutaneous gluteofemoral fat mass is reduced (Cembrowska et al., 2016; Mathew et al., 2016; Stefan et al., 2017; Schulze, 2019; Stefan, 2020; Agius et al., 2023; Valderrabano et al., 2023), possibly yielding an increased waist-to-hip ratio (WHR) with respect to healthy individuals (Eckel et al., 2015; Valderrabano et al., 2023). Their increased central/ectopic fat mass at the expense of the gluteofemoral fat deposits would favor the release of cytokines, adipokines, and non-esterified fatty acids (NEFA) into the circulation, triggering IR and an inflammatory response in the liver and, through intra-myocellular fat accumulation, also in the skeletal muscle. In the long term, this implies hyperinsulinemia, hyperglycemia, dyslipidemia, and loss of skeletal muscle mass (Agius et al., 2023), expectedly leading to reduced muscular strength, as in the metabolic syndrome (Garcia-Hermoso et al., 2020). At the same time, NEFA damages the endothelium of blood vessels, and MUHNW patients later develop hypertension and cardiovascular disorders, as in the metabolic syndrome (Ruderman et al., 1998; Echouffo-Tcheugui et al., 2019). Because the metabolic syndrome is accompanied by dental/periodontal damage (Timonen et al., 2010) coupled with an increase in the circulating levels of FGF21 (Sawangpanyangkura et al., 2022), which can be potentially cariogenic (Soberg et al., 2017), and MUHNW subjects express IR, inflammation, and later progression to dysmetabolism like in the metabolic syndrome, it is reasonable to expect disorders of their dental health as an early event. Finally, the MUHNW phenotype is more frequently encountered in males, and its prevalence is estimated at approximately 30% of the Caucasian population (Mathew et al., 2016; Agius et al., 2023); thus, MUHNW males would be ideal for *in vivo* testing of MYO effects on dental health.

Starting from a population screening (link at https://www.centromedicogalliera.com/progettoosteonet-comunedigalliera-universit%C3%A0diparma), we found that 5.2% of the subjects were young males (age ≤45 y.o.) of average healthy BMI but with body composition and energy expenditure consistent with that of MUHNW patients. They were characterized by a mean fat mass percentage (FM%) of more than 22%, as derived by truncal plicometry (Tresaco et al., 2009), which is a cut-off value of MUHO in Indo-European populations (Ho-Pham et al., 2011; Tayefi et al., 2019). As truncal plicometry evaluates truncal fat, we concluded that our sample was characterized by an accumulation of truncal fat, as expected in a MUHNW phenotype (Klitgaard et al., 2020; Valderrabano et al., 2023). Compatible with IR of a MUHNW phenotype, muscle strength adjusted to body weight or normalized grip strength (NGS) resulted lower than 0.615, which is a typical cut-off value by more than 30% of young males of normal weight having laboratory features of metabolic syndrome (Garcia-Hermoso et al., 2020). Consistently, their mean amount of Fat Free Mass (FFM) adjusted to the height or FFM index (FFMI) was lower than that recorded for young males with IR measured by the surrogate fasting index HOMA-IR, and it expressed the reduced muscle mass of sedentary MUHNW subjects (Ghachem et al., 2019).

All the subjects had negligible plaque, malocclusion, and enamel defects, leading to the exclusion of these factors as cariogenic (Silness and Roynstrand, 1985; Sa-Pinto et al., 2018; Nota et al., 2020; Bernhardt et al., 2021). However, they had a mean of more than three occlusal caries/patient. In contrast, we did not find a specific distribution of caries in relation to the hemiportions of the jaw and maxilla, which is consistent with the absence of enamel and/or masticatory disorders (Demirci et al., 2010). This evidence supported the hypothesis that IR caused early susceptibility to the cariogenic effect for those dental areas that are more readily exposed by their anatomy to pH changes and pathogens, such as the occlusal surfaces (de Paiva et al., 2018). Finally, we did not rule out genetic predisposition to dental caries, which account for approximately 65% of the Caucasian population (Shaffer et al., 2015), nor did we avoid a sex bias related to the possibly less cariogenic salivary pattern in males with respect to females (Galvao-Moreira et al., 2018). However, the young age of the subjects reduced the confounding effect of aging associated with the other types of caries (Gati and Vieira, 2011).

#### 4.1.3 In MUHNW males, the predicted IR correlates with the number of dental caries

In our subjects, we found a direct, statistically significant correlation between the number of occlusal caries and more than 60% of the anthropometric indexes predicting IR in relation to the truncal and gluteofemoral fats (BMI, WC, WHR, WtHR, and BRI; for abbreviations, see 2.1.2.). An increase in the value of these parameters has been linked to an increase in IR and the risk of atheromatosis in young adult males of different ethnicities (Greenlund et al., 1999; Koskinen et al., 2009; Vasques et al., 2009; Simarro Rueda et al., 2011; Porchia et al., 2014; Gonzalez-Cantero et al., 2018; Lechner et al., 2021), which is also in the absence of direct measurements of insulin and glucose such as HOMA-IR, with the latter providing only a weak prediction if considered as a single parameter (Fox et al., 2017). Consistent with these results, the number of occlusal caries displayed a statistically significant inverse correlation with the energy expenditures (REE and TEE; for abbreviations, see 2.1.2.) adjusted to the indexes of visceral fat (BMI, WC, and BRI). This indicated that the higher the amount of visceral fat, the lower the metabolic activity, as observed in metabolic syndrome (Buscemi et al., 2007; Pujia et al., 2016). A similar result was also achieved with anthropometric indexes of fat-free mass and skeletal muscle strength. Specifically, a direct and statistically significant correlation was observed between the number of occlusal caries and indexes of striated muscle mass (FFMI, handgrip strength, and NGS; for abbreviations, see 2.1.2.), as in the presence of IR/metabolic syndrome (Ghachem et al., 2019; Garcia-Hermoso et al., 2020; Zaniqueli et al., 2021; Lagace et al., 2022). In contrast, an inverse statistically significant correlation occurred when the energy expenditures (REE and TEE) were adjusted to FFMI and handgrip strength, suggesting that the higher the skeletal muscle mass, the lower the metabolic activity, which is a finding in agreement with the possibility that MUHNW subjects have a lower energy expenditure with respect to controls (Klitgaard et al., 2020).

Accordingly, a direct statistically significant correlation occurred between the number of occlusal caries and the composite score of anthropometrically predicted IR. A number of indexes considering laboratory values (insulin, glucose, lipids, and inflammatory proteins) have been developed to evaluate IR (Singh and Saxena, 2010; Garcia-Hermoso et al., 2020; Park et al., 2021; Gastaldelli, 2022), with the intent to maximize the contribution of as many variables as possible related to IR. Our composite score (for details, see 2.1.2.) was developed in the same perspective, but focusing on the body composition (adjusted to the sample variance), which is a well-established expression of IR (Vasques et al., 2010; Piqueras et al., 2021). This was done with the intent to speed up outpatient selection and reduce laboratory costs in light of a forthcoming clinical trial. It confirmed that tooth decay was recorded in a likely MUHNW phenotype and supported the assumption that IR *per se* was a causal factor for the cariogenic outcomes.

### 4.2 *In vitro* proof-of-concept

#### 4.2.1 MYO stimulates replication of vascular and stromal cells

In reverting *in vivo* (bed) evidence of dental IR treatable with MYO to an *in vitro* (benchside) scenario, we set a proof-of-concept searching for an insulin-mimetic effect of MYO on the growth of vascular endothelial cells (HUVECs) and embryonic fibroblasts. These are recognized surrogates of vascular and MSC-like cells of the dental pulp and alveolar bone (Huang et al., 2006; Sun et al., 2011; Dissanayaka et al., 2012; Teng et al., 2012; Dissanayaka et al., 2015) that, in turn, represent the largest pulpar/alveolar cell populations (Gaje and Ceausu, 2020; Alvarez-Vasquez and Castaneda-Alvarado, 2022; Ren et al., 2022), have very similar molecular signatures in pulp and periodontium, including alveolar bone (Pagella et al., 2021), and respond to insulin and IGFs through the same receptor (Wang et al., 2012; Lauritano et al., 2015; Escudero et al., 2017; Oyanagi et al., 2019; Bashir, 2021; He et al., 2022; Huseynova et al., 2022), which are also in the course of dental caries (Alkharobi et al., 2018). Consistently, IR leads to a microangiopathy in terminal arteries of the dental pulp and in periodontal/alveolar vessels, pulpar fibrosis with loss of fibroblasts, and altered pulpar nerve sensitivity (Bender and Bender, 2003; Fenn et al., 2019; Puscasu et al., 2021). This latter sensory disturbance is strictly dependent on the pulpar microcirculation that is organized as a blood–brain barrier without lymphatics and intermingled with glial-like sensory cells abutting odontoblasts and dentin (Farahani et al., 2011), stressing the crucial role of vascular cells to obtain an *in vivo* physiological regeneration of the dental/alveolar complex. Thus, vascular endothelial cells and fibroblasts of the dental pulp and/or alveolar bone are expected to be primary targets of MYO *in vivo*.

Using cell monolayers, we showed that MYO was able to increase the proliferation of HUVECs at a statistically significant level with respect to untreated controls in a concentration range between 40 and 640 microM and after 8 days of treatment. No changes to the classical morphology of these endothelial cells (Jaffe et al., 1973) occurred for the entire period of culture and with all MYO concentrations. A growth effect of MYO on vascular cells had already been observed in an *in vitro* study attempting to counteract the inhibitory effect of diabetic hyperglycemia on the replication of endothelial cells (Lorenzi and Toledo, 1986). In that study, responses were in a concentration and time range consistent with our current results, and similar to our reverse bed-to-benchside approach, the authors started from the *in vitro* reproduction of an *in vivo* clinical evidence (the diabetic hyperglycemia *in vitro* mimicked with corresponding glucose concentrations) and *in vitro* tested the supposed *in vivo* capacity of MYO to counteract the hyperglycemic inhibition on vascular cell growth. Likewise, we started from the *in vitro* reproduction of blood levels of MYO following its *in vivo* administration (2–6 g/day) to treat IR (Groenen et al., 2003; Montanino Oliva et al., 2018; Garzon et al., 2019) and *in vitro* tested the supposed *in vivo* capacity of MYO to counteract IR on vascular cell turnover, obtaining a remarkable cell growth response.

Because diminished number of fibroblasts characterizes the fibrotic changes of carious lesions (Balic et al., 2023), we endorsed an experimental model and MYO concentration range corresponding to that of HUVECs for studying the effect of MYO on stromal cells. Using cell monolayers, we showed that MYO was able to increase the proliferation of Swiss NIH3T3 embryonic fibroblasts to a higher statistically significant level with respect to untreated controls selectively at a concentration of 80 microM, the only one ineffective on HUVEC replication. However, similar to HUVECs, MYO growth action did not affect fibroblastic morphology, suggesting that MYO did not interfere with the state of differentiation of embryonic fibroblasts; however, it favored their turnover. The capacity of MYO to *in vitro* counteract growth-inhibitory effects of hyperglycemia on human skin fibroblasts had previously been observed in a concentration range consistent with that in our current results, whereas higher concentrations were devoid of any effect (Sibbitt et al., 1989). Thus, it was clearly demonstrated that the mitogenic response of human fibroblasts to MYO occurred in a well-determined concentration range, as in our experiments. This conclusion was reinforced by the *in vitro* capacity of MYO to exert toxic-like/apoptotic-like effects on rat bone marrow MSCs in the short term (36-h) only at concentrations equal to or above 360 microM (Olyai et al., 2017) and by *in vivo* evidence that MYO stimulates growth of mouse mandibular but not tibial plate chondrocytes (Yamaguchi et al., 2019), confirming that its trophic action may vary in relation to the concentration and type of mesodermal cell tested. As Swiss NIH3T3 cells have a number of features similar to multipotent MSCs (Dastagir et al., 2014), and multipotent MSCs share this property with dental pulp and alveolar bone stem cells (Sloan and Smith, 2007; Huang et al., 2010; Demarco et al., 2011; Moreno Sancho et al., 2019; Mozaffari et al., 2019; Ahmed et al., 2020; Fu et al., 2022; Guy, 2022; Jafer et al., 2022; Li et al., 2023), MYO results in a trophic factor sufficient to influence dental/alveolar/oral bone regenerative processes through resident stem cells in concentration- and site-specific manners.

#### 4.2.2 Signaling systems involved in the growth response of vascular endothelial and stromal cells to MYO

As expected, a key molecular switch in the proliferative action of MYO on endothelial cells *in vitro* is the PI3K/serine/threonine kinase Akt (Michell et al., 1981; Berridge et al., 1983; Simioni et al., 2018; Tuncel and Kalkan, 2018), which is a major signaling pathway activated by insulin and IGFs. In turn, PI3K can bind Ras and cross-talk with the MAPK cascade, the primary mitogenic pathway activated by insulin and IGFs (Escudero et al., 2017; Hakuno and Takahashi, 2018). Consistently, a recent *in vitro* study on HUVECs showed that 1 mM MYO was able to induce rapid phosphorylation of Akt and MAPK (D’Oria et al., 2017), confirming its insulin mimetic action on endothelial cells via converging pathways. As IR leads to the inactivation of the PI3K/Akt/mTOR signaling pathway (Li et al., 2022; Ramasubbu and Devi Rajeswari, 2023), the *in vitro* growth effect of MYO on endothelial cells predicts its capacity to counteract the detrimental effects of *in vivo* IR on vascular cells (Budi et al., 2019; Jiang et al., 2003; D’Oria et al., 2017), including the dental/alveolar complex. In particular, bypassing the inhibition on the vasodilatory PI3K/Akt/nitric oxide pathway, MYO can rebalance the hyperactivation of the Ras/MAPK pathway, which otherwise leads to hypersecretion of the vasoconstrictor endothelin. This mechanism is similar to that of antidiabetic drugs that activate the PI3K pathways via the release of adiponectin, such as thiazolidinediones (Kim et al., 2006). Finally, by means of its insulin-mimetic property, MYO should be able to stimulate the expression of cell surface receptors for TGFbeta, which are necessary for TGFbeta/Akt-mediated endothelial migration and differentiation during revascularization (Hata and Chen, 2016; Budi et al., 2019).

A very similar array of transduction cascades is likely to come into play with the *in vitro* growth response of fibroblasts/MSC-like cells to MYO. Indeed, the activation of PI3K/Akt signaling chains has been shown to induce mobilization, renewal, and differentiation of pulpar and alveolar stromal cells (fibroblasts and MSCs), and formation of mineralized dentin (Tanaka et al., 2018; Ramazzotti et al., 2019; Zhang et al., 2020), which is a mechanism compatible with the molecular signaling of MYO. In addition, pulpar stem cells and odontoblasts may be mobilized by molecules activating the PI3K/Akt/mTOR and Wnt/beta catenin signaling pathways, leading to the formation of reactionary/reparative dentin (Li et al., 2017; Neves et al., 2017; Neves and Sharpe, 2018). As these mitogenic cascades have the potential to cross-talk (Precilla et al., 2022), MYO would offer the unique opportunity to simultaneously activate multiple information lines for dental cell growth. Expectedly, stem cells from the periodontal ligament and MSCs of the alveolar and masticatory bones might equally respond to MYO with regeneration of the alveolar and surrounding bone tissue (Ramazzotti et al., 2019; Zhang et al., 2020; Fu et al., 2022). Human pulpar MSCs have been shown to differentiate to osteoblasts through activation of the energy sensor AMPK and ensuing Akt/mTOR signaling (Pantovic et al., 2012), a transduction chain well known to be activated by MYO (Cabrera-Cruz et al., 2020).

### 4.3 *In silico* clinical outcome pathway

Using information analysis, we substantiated the predicted link between *in vitro* regenerative actions of MYO and *in vivo* evidence of its suitability to counteract tooth decay in a selected group of patients with IR. To achieve this, we were able to take advantage of a recent trend of bioinformatic studies focused on providing knowledge tools for reliably transferring an effect/action recorded in a specific experimental domain (e.g., the *in vitro* trophic effect of MYO) to another experimental domain (e.g., the *in vivo* capacity of MYO to stimulate tissue/organ growth, including dental hard tissues and oral bones). This approach gives rise to an oriented graph of events depicting the different theoretical/*in silico*, *in vitro*, and *in vivo* key steps for reaching a clinically relevant action of the molecule studied, progressing through well-determined steps of its recorded and published actions, that is, a molecular initiating event followed by key events, and a final clinical outcome. Collectively, this chain specifies a clinical outcome pathway or COP (Korn et al., 2022). It stems from the widely used toxicological approach of adverse outcome pathway or AOP, generating the network of knowledge events involved in the toxic effects of a molecule, from its *in silico* to its *in vivo* levels (Spinu et al., 2020). Like AOP, the reliability of the knowledge chain and conclusions of a COP can be quantified using a number of information theory and probabilistic Bayesian approaches that provide increasing levels of confidence in the outcome (Spinu et al., 2020; Moe et al., 2021).

In our analysis, we used two different and complementary techniques; in the first phase, we built up an *ad hoc* database on MYO trophic effects extracted from the relevant international literature by manual curation and focused on the molecular knowledge (signaling systems and transduction cascades), leading to *in vitro* mitogenic effects, as well as *in vivo* growth responses in animal and human organ models, including our current results. This is a well-established technique to generate a precise dataset aimed at building a biomedical knowledge graph (Nicholson and Greene, 2020). Then, we quantified informative strength of each categorical variable targeted inside each node of the graph (i.e., the descriptor of the MYO action at that level) from the molecular to real-word levels. This was obtained by computing the weight of evidence and information value (I.V.) of each node, as suggested for quantification of AOP (Collier et al., 2016; Spinu et al., 2020). For the entire chain of cause–effect events, we obtained a cumulated I.V. depicting a high predictive power (>0.5), which indicated high reliability of the credence for a cell regenerative action of MYO (details of the procedure are given at 2.3.1.). In the second phase, to reduce/exclude the bias possibly intrinsic to a very high I.V., we searched for the same chain of cause–effect elicited by MYO but throughout different levels of biological organization and phylogenetic scale, from lower vertebrates to humans. This is an established strategy to corroborate in an unbiased manner the information extracted from a database used for building a biomedical knowledge graph with predictive purposes (Nicholson and Greene, 2020). Remarkably, the transfer of knowledge of MYO trophic actions (related to the nodal descriptors) to different taxonomic levels supported the idea that MYO may effectively act as a regenerative factor on a wide tissue/organ array in mammals and man, thus making it reasonable to expect its capacity to counteract the detrimental effect of IR on *in vivo* tooth decay.

### 4.4 Limitations of our study

Our results are limited by the lack of a placebo-controlled clinical trial in insulin-resistant, MUHNW subjects. This would show the utility of the insulin-sensitizing/insulin-mimetic action of MYO to induce repair/regeneration in their dental/oral bone district. To empower the specificity of the results, the trial could be extended to different age groups and ethnicities (Mathew et al., 2016; Agius et al., 2023; Ye et al., 2023), and the number and type of clinical variables under scrutiny could be increased, including HOMA-IR/QUICKY indexes, liver elastrography score, lipid profile, inflammatory proteins, and blood pressure. In this manner, insulin-resistant target subgroups could be obtained, as requested by the principles of stratified medicine and analysis of homogenous patient clusters for testing new therapeutic factors.

### 4.5 Clinical implications of our study including tissue engineering

Several patients’ groups could potentially benefit from the insulin-mimetic action of MYO. These include subjects with poor dental health caused by iatrogenic, post-traumatic, infectious, and/or malnutritional disorders, leading to the inhibition of cell turnover in the dental/alveolar complex, and surrounding oral bones, in particular, patients with medication-mediated osteonecrosis of the jaw (Vescovi et al., 2014; Ghidini et al., 2017; Campisi et al., 2020), cancer and aging (Gati and Vieira, 2011; Di Fede et al., 2018), and rare osteometabolic disorders such as trico–dento–osseous syndrome (TDO; OMIM #190320), Stormorken syndrome (STRMK; OMIM #185070), and related tubular aggregate myopathy (TAM; OMIM #160565 and #615883).

In all these patients, MYO might favor osteoblastic differentiation of MSCs through the intermediate filament desmin (DES) (Di Conza et al., 2023). DES is expressed in resting human dental pulp stem cells (Karaoz et al., 2010), becomes hyperexpressed during osteodifferentiation in dental ligament fibroblasts co-cultured with endothelial cells (Neeley et al., 2010), and needs to maintain a dephosphorylated state for its cytoskeletal functioning (Inagaki et al., 1988). As *in vitro* and *in vivo* DES dephosphorilation is ensured by PI3K/Akt-dependent inhibition of GSK3beta (for a review, see Agnetti et al., 2022), and inhibitors of GSK3beta *in vivo* stimulate turnover of pulpar stem cells and odontoblasts (Li et al., 2017; Neves et al., 2017; Neves and Sharpe, 2018), the capacity of MYO to activate the PI3K/Akt signaling might result in the deactivation of GSK3beta and rescue of DES filaments, thus favoring alveolar/mandibular regenerative osteogenesis, which also occurs in conditions of IR. DES may act as a cytoskeletal controller of intracellular Ca^2+^, preventing intracellular hypercalcemia while ensuring adequate extracellular Ca^2+^ for mineral deposition (Di Conza et al., 2023), and unopposed rise in intracellular Ca^2+^ favors DES depolymerization/inactivation (Agnetti et al., 2022) with defective extracellular mineralization.

Although TDO, STRMK, and TAM may have tooth decay based on reduced enamel mineralization and mechanical resistance (a type of *amelogenesis imperfecta*), defective reparative/tertiary dentin can occur (typically in TDO), giving rise to dental caries, dentoalveolar abscesses, and, in TDO, an enlarged pulpar chamber or taurodontism (Wright et al., 1997; Eckstein and Lacruz, 2018). In particular, different *in vitro* transfections and *in vivo* transgenic mice for mutations of the TDO DLX3 gene downregulate DES expression of MSC-like cells committed to osteoblastic differentiation (Choi et al., 2008), reduce odontoblastic differentiation of pulpar MSCs and dentin mineralization (Zeng et al., 2017), and induce odontoblastic apoptosis and altered dentin structure (Choi et al., 2010). Similarly, the stromal interaction molecule 1 (STIM1) R304W gain-of-function knock-in mouse recapitulates the gain-of-function mutations of STRMK/TAM patients, leading to overactivation of the store-operated Ca^2+^ entry (SOCE) system through dominant STIM1 and Ca^2+^ release-activated Ca^2+^ (CRAC) channel ORAI1 mechanisms (Silva-Rojas et al., 2019; Gamage et al., 2020). This brings about excess Ca^2+^ inflow and cellular stress, believed to be unopposed as a result of damaged DES regulation of cellular Ca^2+^ transit (Zhang et al., 2021). Consistently, mutated mice display enamel defects similar to those of STRMK/TAM patients (Wright et al., 1997; Eckstein and Lacruz, 2018; Silva-Rojas et al., 2019). Therefore, it appears that restoration of an altered DES function might be crucial for the regulation of dental/alveolar/oral bone mineralization in these disorders, and MYO might represent the first molecule of clinical significance to stimulate the DES pathway for mineralized tissues.

Increased risk of tooth decay/periodontitis due to poor oral bone quality and low mass density characterize postmenopausal women. In this context, it is interesting that MYO may exert an anabolic effect on osteoblasts and osteoprogenitors. In particular, the sodium/MYO cotransporter 1 (SMIT1) knockout mouse displays a number of skeletal and osteogenetic abnormalities, including delayed embryonic bone formation, postnatal short limbs, curved vertebral column, drooped skull, reduced bone mineralization, particularly in the cancellous trabeculae, reduced trabecular bone volume and collagen content, diminished cortical bone mineral density, loss of osteoblasts, low number of skeletogenic MSCs, and poor osteoblastic differentiation of these MSCs with reduced expression of Runx2, trascription factor Sp7 (osterix), and osteoblastogenetic (but also osteoclast-inhibitory) regulator NFATc1. All these defects were substantially removed in pups by dietary administration of MYO, both prenatally to nursing dams and postnatally after weaning (Dai et al., 2011). Interestingly, inositol triphosphate (IP3) concentrations in differentiating SMIT1-knocked out with respect to wild-type MSCs, and following administration of MYO to reactivate their osteogenetic fate, showed no significant difference between the two cell types (Dai et al., 2011). This suggests that the bone anabolic effects of MYO did not primarily involve a PLC-mediated signaling chain (Berridge, 1993) but likely depended on its conversion to the PI3K/Akt/mTOR pathway. Consistently, *in vivo* supplemental administration of MYO had previously been shown to increase the radioactive calcium uptake in rat bones (Angeloff et al., 1977).

In contrast, the MYO isomer D-chiro-inositol was shown to *in vitro* inhibit rodent osteoclastogenesis (Liu et al., 2012; Yu et al., 2012), whereas the D-chiro-inositol analog, D-pinitol, *in vivo* increased bone mineral density (primarily cancellous) in diabetic rats (Liu et al., 2023). Thus, these two MYO derivatives would exert a pre-eminent bone anti-resorptive action, which is similar to the anti-resorptive effect of the polyphosphated forms of MYO, whose biphosphonate-like mechanism was observed *in vitro* to prevent bone resorption in organotypic rat bone cultures (Gomes et al., 1984) and *in vivo* to maintain/recuperate bone mass density in humans (Lopez-Gonzales et al., 2008; Lopez-Gonzales et al., 2011; Lopez-Gonzales et al., 2013; Sanchis et al., 2023). As a combination of MYO with D-chiro-inositol is currently used to reduce insulin resistance in PCOS (for a review, see Unfer et al., 2012), this combination might also prove useful as an anabolic/anti-resoptive treatment in osteoporotic tooth decay/periodontitis (Suri and Suri, 2014; Lee and Myong, 2022).

In general, then, MYO could clinically be useful to trigger regeneration of alveolar bone in the extraction sockets during osteointegration of dental implants, a process at risk for inflammation (per-implantitis) and physical matching between natural and synthetic materials (Moreno Sancho et al., 2019; Fu et al., 2022; Jafer et al., 2022).

Finally, a number of tissue engineering techniques for dental hard tissues and oral bone might find in MYO a new molecule for the amelioration of their reparative/regenerative action. In particular, we expect that MYO would electively target vascular and stromal/MSC-like cells of the dental/alveolar/oral bone tissues. As regeneration in these areas is always strongly related to the extent and functionality of the local revascularization (Demarco et al., 2011; Dissanayaka et al., 2012; Dissanayaka et al., 2015; Athirasala et al., 2017; Mozaffari et al., 2019; Ahmed et al., 2020; Fu et al., 2022), the vasculotropic actions of MYO would add new momentum to the medical products for translational dentistry and craniofacial medicine (Bakopoulou, 2020). MYO might also be easily delivered locally, through release from implanted biocompatible scaffolds, to promote recruitment of resident and neighboring stromal/MSC-like progenitors, as recently shown for other molecules in chemotaxis-induced cell homing (Kim et al., 2010). Other advantages of MYO would be its low cost, readiness to be delivered in appropriate amounts for the entire duration of repair/regeneration, its reliability and repeatability of action, and the high clinical tolerability. In contrast, all the recombinant and biologically active growth factors, congener molecules, mineralized complexes, and anti-resorptive drugs tested up to now as possible inducers of repair/regeneration at different pulpar/periodontal/alveolar/oral bone levels (Sloan and Smith, 2007; Huang et al., 2010; Demarco et al., 2011; Song et al., 2017; Moreno Sancho et al., 2019; Mozaffari et al., 2019; Ahmed et al., 2020; Yao et al., 2020; Yao et al., 2020; Cao et al., 2021; Xie et al., 2021; Fu et al., 2022; Guy, 2022; Jafer et al., 2022; Vaquette et al., 2022; Wang et al., 2022; Ariano et al., 2023; Li et al., 2023) are missing one or more of the advantages of MYO (Mitsiadis and Rahiotis, 2004; Moreno Sancho et al., 2019; Faggion, 2020; Sharpe, 2020; Jafer et al., 2022; Li et al., 2023).

## 5 Conclusion and future perspectives

By combining *in vivo*, *in vitro*, and *in silico* studies, we concluded that MYO has the potential to be an effective, low cost, easy to deliver, and highly tolerable regenerative factor in dentistry and oral surgery. It may simultaneously activate different growth signaling cascades elective for the dental/Nalveolar/oral bone system, primarily the insulin chain. As such, it could be used to treat/prevent dental/oral bone decay in both IR-related disorders and, through its insulin-mimetic action, also in other dysmetabolic and destructive/necrotic processes of the dental/maxillo-facial context. Finally, it might profitably be used in tissue engineering of hard/mineralized tissues, contributing to ameliorate personalized regenerative dentistry and oral surgery.

## Data Availability

The raw data supporting the conclusion of this article will be made available by the authors, without undue reservation.
